# Artificial sweetener intake and risk of chronic obstructive pulmonary disease: a mediation analysis based on plasma metabolic biomarkers

**DOI:** 10.1186/s12986-026-01142-z

**Published:** 2026-07-07

**Authors:** Miaomiao Fan, Yan Chen, Xiaolin Yin, Shasha Zhao, Jian Feng, Zhaoping Li, Qihui Zhang, Chen Jiang, Hao Bai, Liyong Chen

**Affiliations:** 1 Department of Nutrition, Qilu Hospital of Shandong University, Shandong Jinan, China; 2 Department of Toxicology and Nutrition, School of Public Health, Cheeloo College of Medicine, Shandong University, Shandong Jinan, China; 3 Department of Health, Shandong University of Traditional Chinese Medicine, Shandong Jinan, China; 4 Clinical Epidemiology Unit, Qilu Hospital of Shandong University, Shandong Jinan, China; 5 Department of Clinical Nutrition, Shandong Provincial Hospital Affiliated to Shandong First Medical University, Shandong Jinan, China

**Keywords:** UK Biobank, Chronic obstructive pulmonary disease, Artificial sweeteners, Metabolic biomarkers

## Abstract

**Background:**

The long-term respiratory health effects of artificial sweetener consumption remain unclear. This study aimed to investigate the associations of artificial sweetener intake with the risk of chronic obstructive pulmonary disease (COPD) and lung function, and to explore the potential mediating role of circulating metabolites.

**Methods:**

Habitual intake of artificial sweeteners (teaspoon/day) was assessed via multiple 24-h dietary recalls. The multivariate Cox proportional hazards regression was used to analyze the association between AS and risk of COPD, and linear regression was used to analyze associations with forced vital capacity (FVC) and forced expiratory volume in 1 s (FEV_1_). A mediation analysis was conducted to assess the contribution of plasma metabolic biomarkers.

**Results:**

A total of 164,656 participants were included. Over a median follow-up of 14.88 years, 4132 incident COPD cases were identified. Compared with non-consumption, higher consumption of artificial sweeteners was associated with a 27% increased risk of COPD (HR = 1.27, 95% CI: 1.14, 1.40) and decreased lung function, including FVC (β: –39.48 mL, 95% CI: -56.53, -22.43, *p* < 0.001) and FEV_1_ (β: –37.08 mL, 95% CI:-50.91, -23.25, *p* < 0.001). A significant positive dose–response relationship was observed between artificial sweetener intake and COPD risk, and a negative dose–response relationship between artificial sweetener intake and lung function parameters. Mediation analysis identified several key mediators, including lipids within intermediate-density and low-density lipoprotein particles, apolipoprotein B, and polyunsaturated fatty acids. Among these, phospholipids in large LDL showed the highest mediating effect (PM: 8.72%; 95% CI: 4.53%, 19.63%), followed by cholesteryl esters in IDL (PM: 8.68%; 95% CI: 4.78%, 20.58%). Beyond lipoprotein sub-fractions, apolipoprotein B served as a significant mediator (PM: 6.70%; 95% CI: 3.67%, 16.35%). Furthermore, key fatty acids were identified as significant mediators.

**Conclusion:**

Higher intake of artificial sweeteners is associated with an elevated risk of COPD and impaired lung function. These associations are partially mediated through pathways related to lipoprotein metabolism and fatty acid profiles.

**Graphical abstract:**

**Figure:**
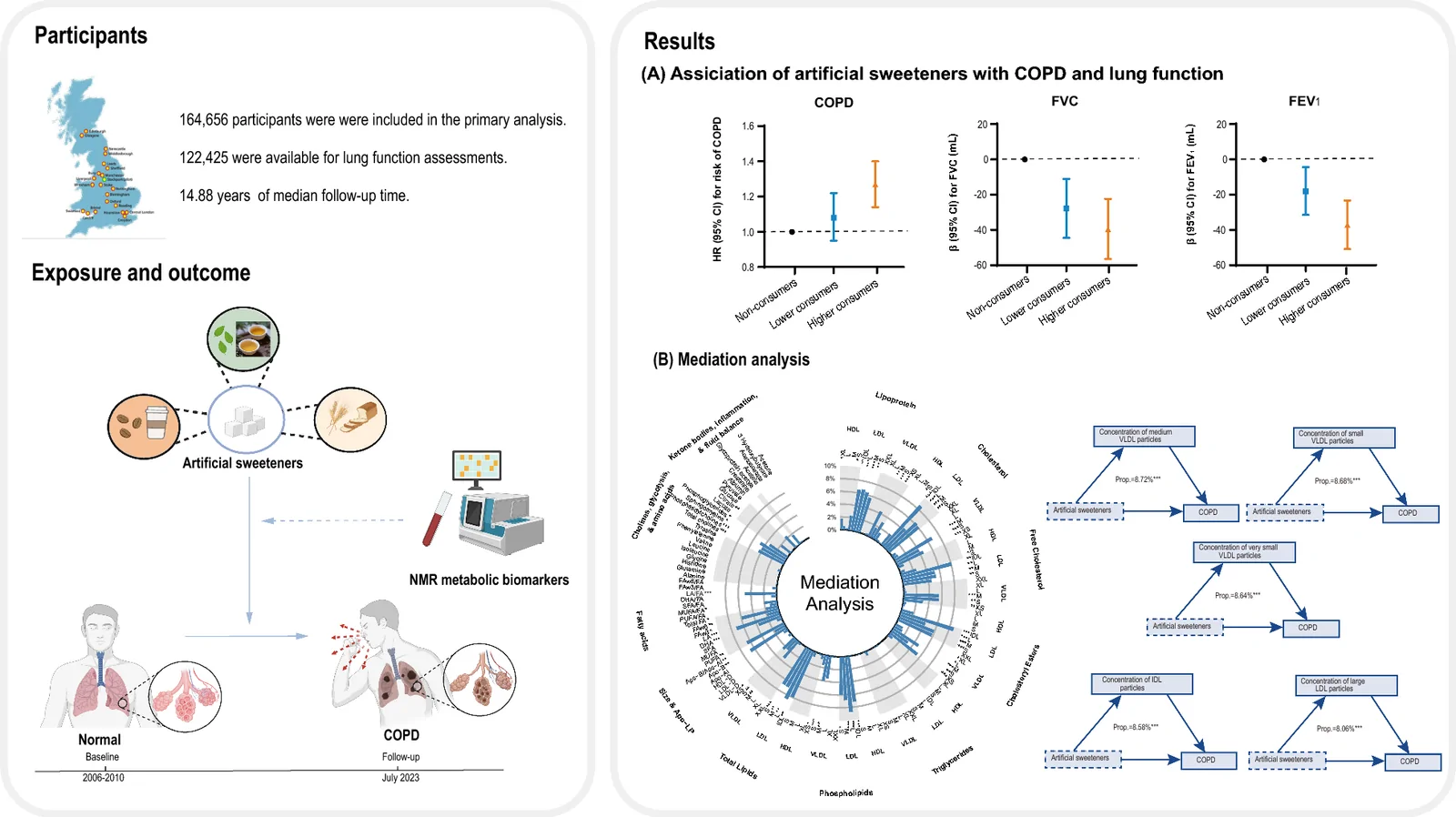

**Supplementary Information:**

The online version contains supplementary material available at https://doi.org/10.1186/s12986-026-01142-z.

## Background

Chronic obstructive pulmonary disease (COPD), characterized by persistent respiratory symptoms and airflow limitation, remains a major global public health challenge [1]. It is currently the third leading cause of death worldwide, resulting in substantial social and economic burdens [2]. Data from the Global Burden of Disease Study 2019 estimated a global prevalence of 212.3 million cases of Chronic Obstructive Pulmonary Disease (COPD). Over the period from 1990 to 2019, the incidence of COPD increased by 86%, while deaths attributable to COPD rose by 30% [3]. The pathophysiology is driven by chronic inflammation that injures the lung parenchyma and airways, sustaining these symptoms and functional deficits [4]. While smoking is established as its primary risk factor [5], a considerable number of COPD cases occur in non-smokers [6], suggesting that other modifiable environmental and lifestyle factors play a crucial role [7–9]. 

Among various lifestyle factors, the relationship between dietary patterns and respiratory health is increasingly gaining attention. As a typical representative of modern industrial food systems, ultra-processed foods (UPFs) are closely associated with an increased risk of various chronic non-communicable diseases, such as obesity, type 2 diabetes, cardiovascular diseases and COPD [10, 11]. Notably, emerging epidemiological evidence has begun to reveal adverse associations between UPFs consumption and respiratory health and has indicated that higher UPF intake is significantly positively correlated with an increased incidence of COPD [12]. Artificially sweetened beverages (ASBs), a prominent category of UPFs, have been independently implicated in impaired respiratory health, such as an elevated risk of asthma and worsening lung function [13]. Moreover, a critical limitation of the existing epidemiological evidence is that this method fails to account for potential confounding by other beverage constituents and ignores tabletop sweeteners—an important exposure source with simpler ingredient profiles. Artificial sweeteners, widely used as sugar substitutes in "sugar-free" beverages and UPFs, primarily include aspartame and saccharin [14]. Although regulatory agencies like the U.S. FDA approve these sweeteners as safe based on short-term studies, accumulating evidence suggests that long-term intake is associated with adverse metabolic outcomes, including increased visceral adiposity and metabolic syndrome [15, 16]. COPD is characterized by disruptions in multiple metabolic pathways, including aberrations in amino acid metabolism, lipid processing, and increased oxidative stress [17–19]. Similarly, experimental studies, particularly on aspartame, have documented its capacity to induce remarkably similar disturbances, including impaired glucose regulation, perturbed lipid metabolism, promoted lipid peroxidation, and dysregulated adipokine signaling [20–22]. However, the translation of these experimental findings to human health outcomes, particularly in the context of COPD, is not fully established and needs further investigation in large human cohorts. Performing mediation analysis on factors linked to both COPD and its risk factors, such as artificial sweeteners, could elucidate potential mechanistic pathways. The UK Biobank, with its extensive data on plasma metabolites and inflammatory markers, presents a valuable resource to facilitate such analyses.

Utilizing data from the UK Biobank, this study assessed the relationships between artificial sweetener consumption, lung function decline, and COPD incidence. We propose the hypothesis that the adverse effects of artificial sweeteners on respiratory health are mediated through their influence on biomarkers. A mediation analysis was therefore conducted to investigate the role of these biomarkers. 

## Methods

### Study population

This investigation utilized data obtained from the UK Biobank, a large-scale prospective study that enrolled more than half a million individuals aged 40 to 69 from 2006 to 2010. Electronic informed consent was secured from every participant. Further details of the study design and methods are described in the Supplementary Methods.

### Ascertainment of COPD and lung function

Prevalent COPD cases at baseline were determined through a combination of self-reported physician diagnoses and prior hospital records. Incident cases of COPD-related hospitalizations and mortality during follow-up were identified from national health and death registries. Diagnoses were defined according to the International Classification of Diseases, Tenth Revision (ICD-10), using code J44. The duration of follow-up was calculated from the date of enrollment until the first occurrence of COPD, death, or July 1, 2022, whichever came first. At the baseline assessment, pre-bronchodilator spirometry was conducted on eligible participants to obtain measurements. Measurements included forced expiratory volume in 1 s (FEV₁) and forced vital capacity (FVC). These were obtained from two to three maneuvers conducted over approximately six minutes, using a Vitalograph Pneumotrac 6800 spirometer. A third exhalation was omitted if the difference between the first two acceptable maneuvers for FEV₁ and FVC was within 5%. 

### Assessment of artificial sweetener intake

Dietary intake data were collected using the Oxford WebQ (https://www.ceu.ox.ac.uk/research/oxford-webq), a web-based 24-h dietary recall tool designed to capture consumption of common foods and beverages [23]. Participants completed the questionnaire on multiple occasions over a five-year period, and mean values were calculated from available responses to represent habitual intake. Artificial sweetener consumption was specifically assessed through participants’ reports of the number of teaspoons of non-caloric sweeteners added to coffee, tea, and cereal, corresponding to UK Biobank data-field IDs 100,380, 100,500, and 100,910, respectively. To compute total daily artificial sweetener intake, we first quantified consumption frequencies of the relevant food categories. Total coffee consumption was derived by summing daily cups across six mutually exclusive coffee types (instant, filtered, cappuccino, latte, espresso, and other coffee). Similarly, total tea intake was calculated from five tea categories (standard, rooibos, green, herbal, and other tea), while cereal consumption was aggregated from eight cereal varieties (porridge, muesli, oat crunch, sweetened cereal, plain cereal, bran cereal, whole-wheat cereal, and other cereal). The amount of artificial sweetener added to each food category was then multiplied by its respective consumption frequency. Finally, total artificial sweetener intake was determined by summing the sweetener quantities across all three categories, expressed as teaspoons per day, providing a comprehensive measure of tabletop artificial sweetener consumption.

### Assessment of covariates

Based on a review of the existing literature, we identified and adjusted for a comprehensive set of potential confounding factors [12, 24]. These included demographic background, socioeconomic status, lifestyle factors, body measurements, and health history. Demographic data comprised age, gender (male or female), and ethnic background (classified as White, Mixed, South Asian, Black, Chinese, or other). Socioeconomic position was evaluated using both educational attainment (grouped into no relevant qualifications, college or university degree, A levels/AS levels or equivalent, O levels/GCSEs or equivalent, CSEs or equivalent, or others) and the Townsend deprivation index, a geographic indicator of material disadvantage. Lifestyle factors included smoking behavior (current, former, never) and alcohol drinking (never, previous, current). Healthy diet score was constructed based on recommended intake levels of various food groups linked to cardiovascular wellness, emphasizing fruits, vegetables, whole grains, fish, dairy products and vegetable oils, with reduced or no consumption of refined grains, processed or unprocessed meats and sugar-sweetened beverages [25]. Physical activity during leisure time was captured by asking participants about their engagement in various types of activities over the previous four weeks, such as walking for pleasure, light do-it-yourself (DIY) projects, heavy DIY work, strenuous sports, other exercises, or none of the above. For respondents reporting any activity, we recorded how often and for how long they performed it. Activity intensity was assigned standard metabolic equivalent of task (MET) values, defined as the ratio of activity-related energy expenditure to the resting metabolic rate. Following this, overall weekly volume (MET-minutes/week) was determined by multiplying the frequency, duration, and MET value for each activity, then summing the results across all activities. The assigned MET values were as follows: 3.5 for walking for pleasure, 2.5 for light DIY, 5.5 for heavy DIY, 8.0 for strenuous sports, and 4.0 for other physical activities [26]. Furthermore, body mass index (BMI) was derived from measured height and weight using the standard formula: weight (kg)/height (m)^2^. Finally, environmental exposures to air pollutants were adjusted for, including nitrogen dioxide (NO₂), nitrogen oxides (NO_X_), particulate matter with a diameter ≤ 2.5 μm (PM_2.5_), particulate matter with a diameter ≤ 10 μm (PM_10_), and particulate matter with a diameter between 2.5 and 10 μm (PM_2.5–10_), with each pollutant variable incorporated as quintiles or as missing.

### Assessment of the mediating biomarkers

Plasma metabolomic profiling was initiated during the first assessment visit (2006–2010), following informed consent. The first repeat visit took place in 2012–2013, while metabolomic quantification was conducted in two phases: Phase 1 from June 2019 to April 2020 and Phase 2 from April 2020 to June 2022. Metabolomic assays using nuclear magnetic resonance (NMR) spectroscopy were performed by Nightingale Health Ltd across both phases. Detailed procedures for sample collection and metabolite quantification have been described previously [27]. Over 280,000 participants had metabolomic data available, with an additional 16,000 undergoing repeat assessment. The present analysis focused on 143 directly quantified metabolites that cannot be derived from other measures [28], including 104 lipoprotein and lipid measures, 3 glycolysis-related metabolites, 15 fatty acids, 9 amino acids, 5 ketone bodies, 4 choline-containing compounds, 1 inflammatory marker, and 2 fluid balance-related metabolites, to identify potential mediators of the association between artificial sweetener intake and COPD.

### Statistical analysis

Baseline characteristics were stratified according to the median intake of artificial sweeteners. Continuous variables are presented as means and standard deviations (SDs), while categorical variables are expressed as numbers and percentages.

The Cox proportional hazards models were used to examine the association between artificial sweeteners and risk of COPD. Subsequently, linear regression was used to analyze the relationship between artificial sweeteners and lung function (FVC/FEV_1_). Effect estimates were presented as hazard ratios (HRs) or β coefficients with their 95% confidence intervals (CIs). Person-years of follow-up were calculated from baseline until the date of death, diagnosis, loss to follow-up, or the end of the follow-up period, whichever occurred first. Four models were constructed in the primary analysis: a crude model without any adjustments; Model 1 adjusted for age, sex, race (White, Mixed, South Asian, Black, Chinese, Other, or missing), educational attainment (no qualifications, college or university degree, A-level/AS-level or equivalent, O-level/GCSEs or equivalent, CSEs or equivalent, other qualifications, or missing), and Townsend deprivation index (quintiles or missing); Model 2 further adjusted for BMI (< 18.5, 18.5 to < 25, 25 to < 30, ≥ 30 kg/m^2^, or missing), smoking status (never, former, current, or missing), alcohol drinking (never, former, current, or missing), healthy diet (yes, no, or missing), leisure-time physical activity (< 500, 500 to < 1000, ≥ 1000 MET-minutes/week, or missing), family history of lung disease (yes, no, or missing), and diabetes (yes, no, or missing); Model 3 additionally adjusted for air pollutants, including NO_2_ (quintiles or missing), NOx (quintiles or missing), PM_2.5_ (quintiles or missing), PM_10_ (quintiles or missing), and PM_2.5–10_ (quintiles or missing). Furthermore, restricted cubic spline (RCS) models were applied to examine potential dose–response relationships.

We performed stratified analyses by age, sex, educational attainment, Townsend deprivation index, BMI, smoking status, alcohol drinking, healthy diet, leisure time physical activity, diabetes, family history of chronic lung disease, NO_2_, NO_X_, PM_2.5_, PM_10_, and PM_2.5–10_ to assess the association between artificial sweeteners and COPD risk and lung function parameters across different socioeconomic, familial, and environmental exposure subgroups. Furthermore, to examine the robustness of the findings, a sensitivity analysis of the association between artificial sweetener intake and incident COPD risk was conducted, excluding participants with follow-up duration shorter than 3 or 5 years.

Moreover, we conducted a mediation analysis to investigate whether the association between artificial sweetener intake (exposure) and COPD (outcome) could be explained by metabolic biomarkers (mediators). Based on data availability in the UK Biobank, 143 biomarkers were included in the mediation analysis. The mediation model was estimated using a bootstrap procedure with 1,000 repetitions, which comprised two components: the mediator model and the outcome model. The mediator fit was specified as “Mediator ~ Covariates + Exposure”, while the outcome fit was defined as “Outcome ~ Covariates + Exposure + Mediator”. The indirect effect was calculated as the product of the effect of the exposure on the mediator and the effect of the mediator on the outcome. The proportion mediated was computed as the indirect effect divided by the total effect, and its 95%CI was estimated using the bootstrapping method [29, 30].

A two-tailed *P*-value < 0.05 was used for primary analyses. For mediation analysis of 143 biomarkers, we applied the Benjamini–Hochberg false discovery rate (FDR) correction to control for multiple comparisons. Statistical significance was defined as FDR controlled *P* < 0.01. All analyses used R version 4.1.1 (https://www.R-project.org/).

## Results

### Characteristics of the study participants

A total of 164,656 participants were included in this study, with mean age of 56.46 years, and 45.7% were male (Supplementary Figure S1). Table 1 presents the baseline characteristics of the study participants stratified by artificial sweetener consumption categories. The total study population categorized as non-consumers (n = 147,639), lower consumers (n = 8,711), and higher consumers (n = 8,306). Significant differences were observed across groups for most demographic, lifestyle, and clinical characteristics. Participants with higher artificial sweetener consumption were older, more likely to be male, and had a higher prevalence of obesity (BMI ≥ 30 kg/m^2^), previous or current smoking, and diabetes. They also exhibited lower educational attainment, higher socioeconomic deprivation, and a lower proportion of healthy diet adherence. In contrast, no significant differences were observed in air pollutant exposures (NO₂, NOₓ, PM₂.₅, PM₁₀, and PM₂.₅-₁₀) across consumption categories.

**Table 1:** Baseline characteristics of the study population

	Total population (n = 164,656)	Non-consumers (n = 147,639)	Lower consumers (n = 8711)	Higher consumers (n = 8306)	P-value
Age, mean (SD), years	56.46 ± 7.88	56.29 ± 7.88	57.62 ± 7.82	58.21 ± 7.48	< 0.001
Male, n (%)	75,246 (45.70)	67,367 (45.63)	3676 (42.20)	4203 (50.60)	< 0.001
Ethnicity, n (%)					< 0.001
White	156,887 (95.28)	140,730 (95.32)	8168 (93.77)	7989 (96.18)	
Mixed background	995 (0.60)	907 (0.61)	56 (0.64)	32 (0.39)	
South Asian	2368 (1.44)	2034 (1.38)	222 (2.55)	112 (1.35)	
Black	2048 (1.24)	1836 (1.24)	138 (1.58)	74 (0.89)	
Chinese	522 (0.32)	496 (0.34)	17 (0.20)	9 (0.11)	
Other	1204 (0.73)	1060 (0.72)	87 (1.00)	57 (0.69)	
Missing	632 (0.38)	576 (0.39)	23 (0.26)	33 (0.40)	
Educational attainment, n (%)					< 0.001
College or University degree	72,004 (43.73)	66,887 (45.30)	2783 (31.95)	2334 (28.10)	
A levels/AS levels or equivalent	21,221 (12.89)	19,050 (12.90)	1122 (12.88)	1049 (12.63)	
O levels/GCSEs or equivalent	33,201 (20.16)	28,902 (19.58)	2158 (24.77)	2141 (25.78)	
CSEs or equivalent	6602 (4.01)	5693 (3.86)	451 (5.18)	458 (5.51)	
Others	16,918 (10.27)	14,853 (10.06)	1058 (12.15)	1007 (12.12)	
No relevant qualifications	13,902 (8.44)	11,543 (7.82)	1081 (12.41)	1278 (15.39)	
Missing	808 (0.49)	711 (0.48)	58 (0.67)	39 (0.47)	
Socioeconomic deprivation index, mean (SD)	75,246 (45.70)	67,367 (45.63)	3676 (42.20)	4203 (50.60)	< 0.001
Missing	184 (0.11)	156 (0.11)	19 (0.22)	9 (0.11)	
Body mass index, n (%)					< 0.001
< 18.5 kg/m^2^	999 (0.61)	960 (0.65)	28 (0.32)	11 (0.13)	
18.5 to < 25 kg/m^2^	65,215 (39.61)	60,885 (41.24)	2354 (27.02)	1976 (23.79)	
25 to < 30 kg/m^2^	67,921 (41.25)	60,136 (40.73)	3965 (45.52)	3820 (45.99)	
≥ 30 kg/m^2^	30,055 (18.25)	25,252 (17.10)	2336 (26.82)	2467 (29.70)	
Missing	466 (0.28)	406 (0.27)	28 (0.32)	32 (0.39)	
Smoking status, n (%)					< 0.001
Never	93,956 (57.06)	86,105 (58.32)	4445 (51.03)	3406 (41.01)	
Previous	57,545 (34.95)	50,183 (33.99)	3581 (41.11)	3781 (45.52)	
Current	12,716 (7.72)	10,964 (7.43)	657 (7.54)	1095 (13.18)	
Missing	439 (0.27)	387 (0.26)	28 (0.32)	24 (0.29)	
Alcohol drinking, n (%)					< 0.001
Never	5261 (3.20)	4550 (3.08)	362 (4.16)	349 (4.20)	
Previous	4681 (2.84)	4072 (2.76)	249 (2.86)	360 (4.33)	
Current	154,550 (93.86)	138,871 (94.06)	8085 (92.81)	7594 (91.43)	
Missing	164 (0.10)	146 (0.10)	15 (0.17)	3 (0.04)	
Healthy diet, n (%)	29,083 (17.66)	26,580 (18.00)	1366 (15.68)	1137 (13.69)	< 0.001
Missing	3414 (2.07)	3031 (2.05)	170 (1.95)	213 (2.56)	
Leisure time physical activity, n (%)					< 0.001
< 500 MET mins/week	52,679 (31.99)	46,639 (31.59)	3128 (35.91)	2912 (35.06)	
500 to < 1000 MET mins/week	37,604 (22.84)	33,878 (22.95)	1930 (22.16)	1796 (21.62)	
≥ 1000 MET mins/week	66,381 (40.31)	60,306 (40.85)	3111 (35.71)	2964 (35.69)	
Missing	7992 (4.85)	6816 (4.62)	542 (6.22)	634 (7.63)	
Diabetes, n (%)	5550 (3.37)	4103 (2.78)	703 (8.07)	744 (8.96)	< 0.001
Missing	344 (0.21)	288 (0.20)	24 (0.28)	32 (0.39)	
Family history of chronic lung disease, n (%)	22,569 (13.71)	19,888 (13.47)	1330 (15.27)	1351 (16.27)	< 0.001
Missing	2271 (1.38)	1977 (1.34)	144 (1.65)	150 (1.81)	
NO_2_, mean (SD)	26.45 ± 7.72	26.47 ± 7.76	26.34 ± 7.42	26.33 ± 7.31	0.112
Missing	2588 (1.57)	2318 (1.57)	139 (1.60)	131 (1.58)	
NO_X_, mean (SD)	43.27 ± 15.43	43.26 ± 15.44	43.28 ± 15.35	43.45 ± 15.20	0.587
Missing	2588 (1.57)	2318 (1.57)	139 (1.60)	131 (1.58)	
PM_2.5_, mean (SD)	9.91 ± 1.03	9.91 ± 1.03	9.92 ± 0.99	9.92 ± 1.00	0.293
Missing	10,021 (6.09)	9056 (6.13)	487 (5.59)	478 (5.75)	
PM_10_, mean (SD)	16.20 ± 1.89	16.20 ± 1.90	16.21 ± 1.88	16.24 ± 1.87	0.229
Missing	10,021 (6.09)	9056 (6.13)	487 (5.59)	478 (5.75)	
PM_2.5–10_, mean (SD)	6.42 ± 0.88	6.42 ± 0.88	6.42 ± 0.89	6.42 ± 0.88	0.865
Missing	10,021 (6.09)	9056 (6.13)	487 (5.59)	478 (5.75)	

NO_2_: nitrogen dioxide, NO_X_: nitrogen oxides, PM_2.5_: particulate matter with a diameter ≤ 2.5 μm, PM_10_: particulate matter with a diameter ≤ 10 μm

### Association of artificial sweetener intake with risk of incident COPD and lung function

**Fig. 1:**
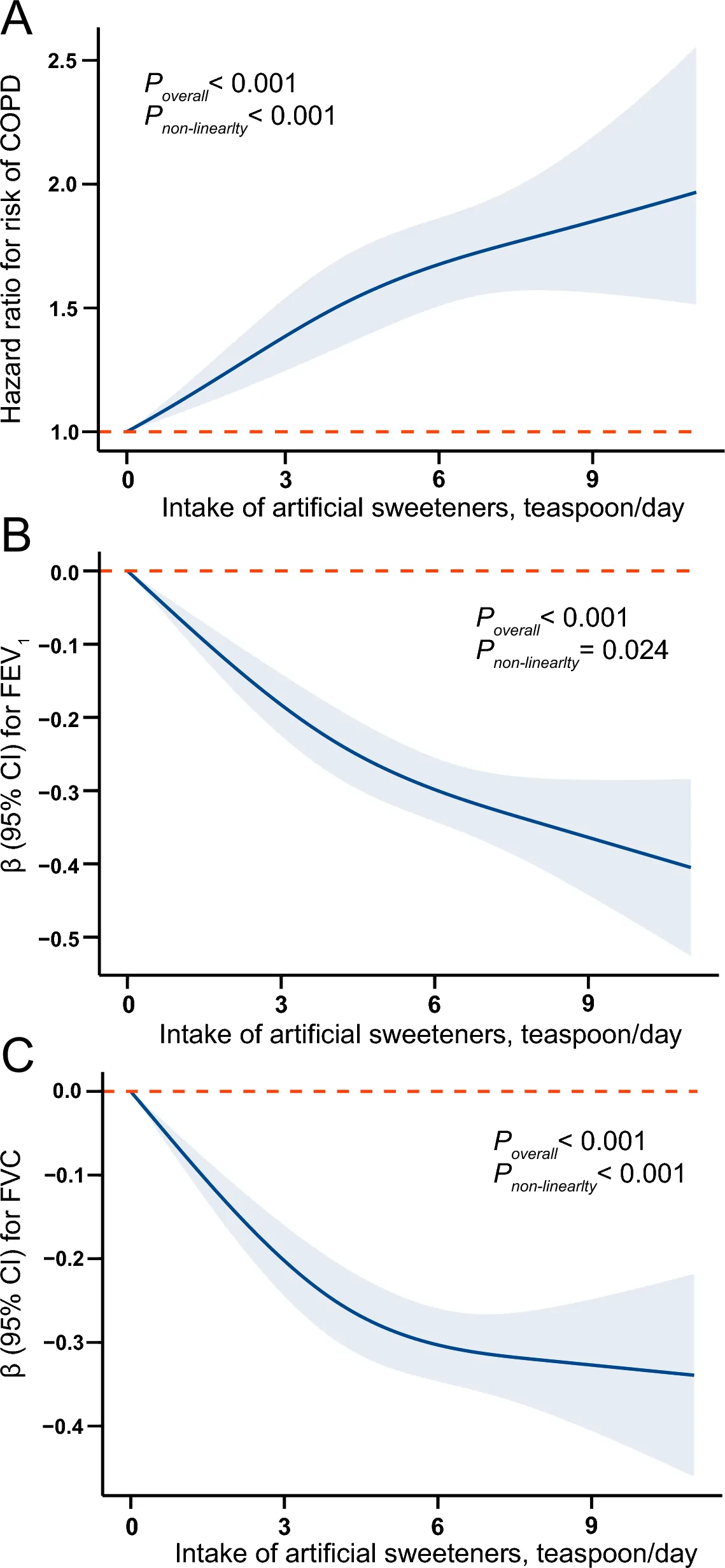
Dose–response associations of artificial sweetener intake with COPD risk and lung function. (**A**) Hazard ratio for COPD risk by daily intake of artificial sweeteners (teaspoon/day). P overall < 0.001, P non-linearity < 0.001. (**B**) Association between artificial sweetener intake and baseline FVC (mL). P overall < 0.001, P non-linearity = 0.024. (**C**) Association between artificial sweetener intake and baseline FEV_1_ (mL). P overall < 0.001, P non-linearity < 0.001

After a median follow-up of 14.88 years (interquartile range [IQR]: 12.51 to 17.15), 4132 individuals developed COPD. Table 2 summarizes the associations between artificial sweetener intake and the risk of incident COPD and lung function decline. In the fully adjusted model, each one-teaspoon increase in artificial sweetener intake was associated with a significantly increased risk of COPD (HR: 1.02, 95% CI: 1.02, 1.03, *p* < 0.001). Compared with non-consumers, higher consumers had a significantly elevated risk of COPD (HR: 1.27, 95% CI: 1.14, 1.40, *p* < 0.001), while lower consumers did not show a significant association (HR: 1.08, 95% CI: 0.95, 1.22, *p* = 0.225). Similarly, higher sweetener intake was associated with reduced lung function, as measured by FVC and FEV₁. In Model 3, higher consumers showed significant declines in FVC (β: –39.48 mL, 95% CI: -56.53, -22.43, *p* < 0.001) and FEV₁ (β: –37.08 mL, 95% CI:-50.91, -23.25, *p* < 0.001) compared to non-consumers. The RCS results demonstrate the significant positive dose–response relationship between artificial sweetener consumption and risk of COPD and the negative dose–response relationship between artificial sweetener consumption and lung function level (Fig. 1).

**Table 2:** Association of artificial sweeteners intake with risk of incident COPD and lung function

	Per 1-teaspoon increase in artificial sweeteners		Non-consumers	Lower consumers		Higher consumers	
	HR/β (95% CI)	P-value	HR/β (95% CI)	HR/β (95% CI)	P-value	HR/β (95% CI)	P-value
COPD							
Events/total	4132/164,656		3418/147,639	287/8711		427/8306	
Crude	1.08 (1.07, 1.09)	< 0.001	1.0 (Ref.)	1.44 (1.28, 1.62)	< 0.001	2.28 (2.06, 2.52)	< 0.001
Model 1	1.05 (1.05, 1.06)	< 0.001	1.0 (Ref.)	1.19 (1.05, 1.34)	0.005	1.68 (1.52, 1.86)	< 0.001
Model 2	1.02 (1.02, 1.03)	< 0.001	1.0 (Ref.)	1.08 (0.96, 1.22)	0.211	1.26 (1.14, 1.40)	< 0.001
Model 3	1.02 (1.02, 1.03)	< 0.001	1.0 (Ref.)	1.08 (0.95, 1.22)	0.225	1.27 (1.14, 1.40)	< 0.001
Lung function parameters							
FVC							
Events/total	2935/122,425		2434/110,033	204/6296		297/6096	
Crude	-13.80 (-16.41, -11.19)	< 0.001	0.0 (Ref.)	-188.07 (-212.73, -163.41)	< 0.001	-136.27 (-161.31, -111.23)	< 0.001
Model 1	-10.30 (-12.10, -8.50)	< 0.001	0.0 (Ref.)	-72.60 (-89.58, -55.62)	< 0.001	-98.57 (-115.85, -81.30)	< 0.001
Model 2	-3.70 (-5.48, -1.93)	< 0.001	0.0 (Ref.)	-27.88 (-44.57, -11.18)	0.001	-39.66 (-56.71, -22.61)	< 0.001
Model 3	-3.69 (-5.47, -1.91)	< 0.001	0.0 (Ref.)	-27.83 (-44.52, -11.13)	0.001	-39.48 (-56.53, -22.43)	< 0.001
FEV_1_							
Events/total	2935/122,425		2434/110,033	204/6296		297/6096	
Crude	-12.29 (-14.33, -10.25)	< 0.001	0.0 (Ref.)	-138.03 (-157.36, -118.71)	< 0.001	-119.74 (-139.36, -100.11)	< 0.001
Model 1	-8.33 (-9.78, -6.88)	< 0.001	0.0 (Ref.)	-43.63 (-57.31, -29.96)	< 0.001	-78.47 (-92.39, -64.56)	< 0.001
Model 2	-3.68 (-5.12, -2.24)	< 0.001	0.0 (Ref.)	-18.08 (-31.62, -4.54)	0.009	-37.12 (-50.95, -23.29)	< 0.001
Model 3	-3.68 (-5.12, -2.24)	< 0.001	0.0 (Ref.)	-17.99 (-31.53, -4.45)	0.009	-37.08 (-50.91, -23.25)	< 0.001

Model 1 was adjusted age, sex, ethnicity (White, Mixed, South Asian, Black, Chinese, other races, or missing), educational attainment (no relevant qualifications, college or university degree, A levels/AS levels or equivalent, O levels/GCSEs or equivalent, CSEs or equivalent, others, or missing), and socioeconomic deprivation index (quintiles, or missing); Model 2 was further adjusted for body mass index (< 18.5, 18.5 to < 25, 25 to < 30, ≥ 30 kg/m^2^, or missing), smoking status (never, previous, current, or missing), alcohol drinking (never, previous, current, or missing), healthy diet (yes, no, or missing), leisure time physical activity (< 500, 500 to < 1000, ≥ 1000 MET mins/week, or missing), family history of lung diseases (yes, no, or missing), diabetes (yes, no, or missing); Model 3 was further adjusted for air pollutants (NO_2_ [quintiles, or missing], NO_X_ [quintiles, or missing], PM_2.5_ [quintiles, or missing], PM_10_ [quintiles, or missing], PM_2.5–10_ [quintiles, or missing])

### Stratified analysis and sensitivity analysis

Based on the stratified analyses presented in Supplementary Tables S1–S3, the association between artificial sweetener intake and incident COPD risk was generally consistent across most subgroups, though some effect modifications were observed. Significant multiplicative interactions were identified for smoking status (P for interaction = 0.010) and leisure-time physical activity (P for interaction = 0.018), suggesting that the association was more pronounced among former or current smokers and those with lower physical activity levels.

A series of sensitivity analyses further supported the robustness of the primary findings. Excluding participants with follow-up duration shorter than 3 or 5 years did not materially alter the results (Supplementary Table S4). The association remained significant after adjusting for multiple covariates, indicating that the observed relationship is not substantially confounded by these variables. We conducted additional sensitivity analyses excluding participants with COPD events occurring within 3 years of follow-up, stratified by obesity status. These analyses showed that the association remained significant in both non-obese and obese subgroups after excluding early events (Supplementary Table S5).

### Mediation analysis

**Fig. 2:**
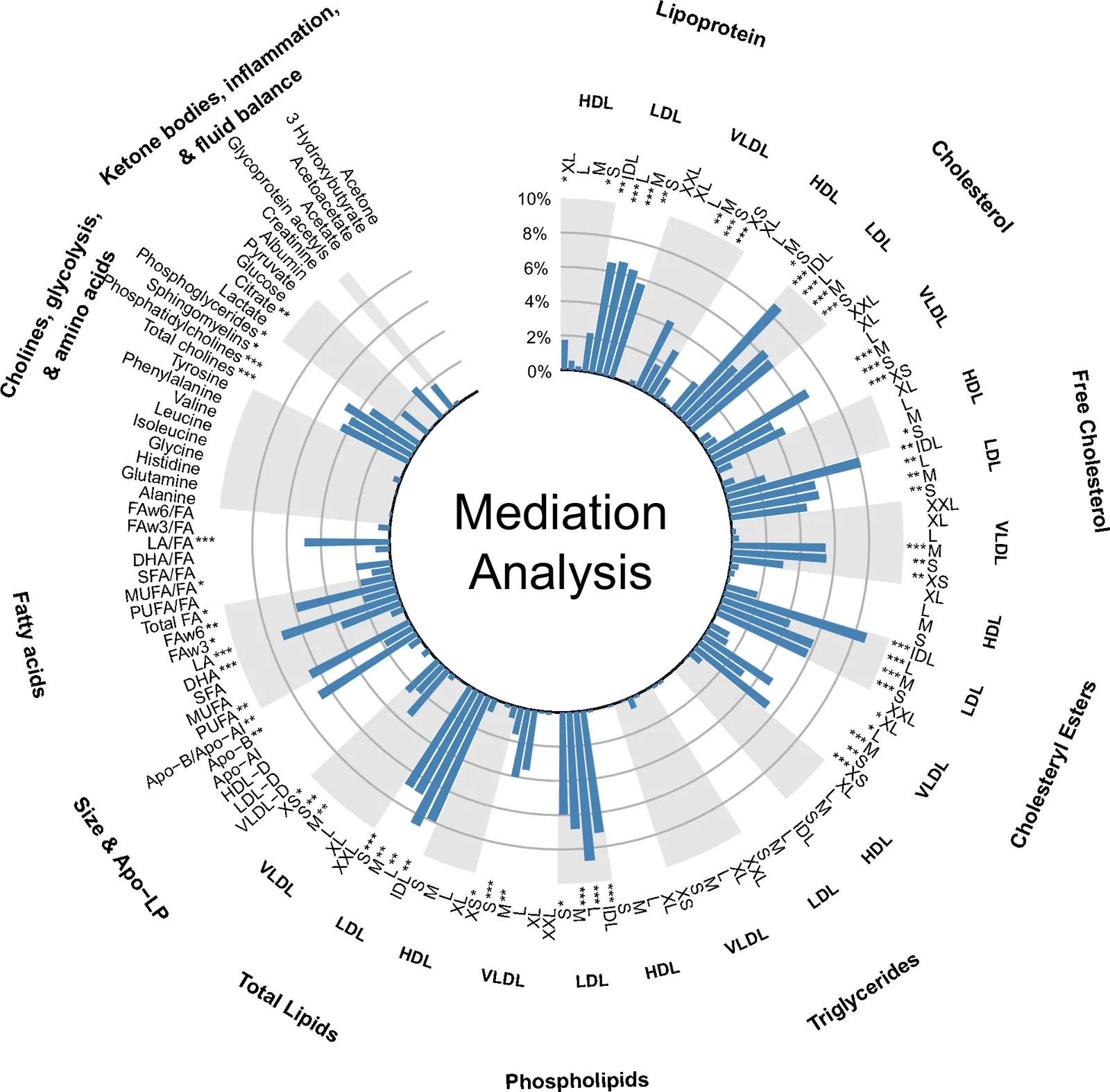
The estimated mediation proportions of metabolic biomarkers on the associations of artificial sweetener intake with COPD risk. Models were adjusted for age, sex, ethnicity, educational attainmen, socioeconomic deprivation index, body mass index, smoking status, alcohol drinking, healthy diet, leisure time physical activity, family history of lung diseases, diabetes, and air pollutants (NO_2_, NO_X_, PM_2.5_, PM_10_, PM_2.5-10_). Asterisks indicate statistical significance (*P < 0.05, **P < 0.01, ***P < 0.001). Apo, apolipoprotein; DHA, docosahexaenoic acid; FA, fatty acids; acid; HDL, high-density lipoproteins; IDL, intermediate-density lipoproteins; L, large; LA, linoleic acid; LDL, low-density lipoproteins; LP, lipoprotein; M, medium; MUFA, monounsaturated fatty acids; PUFA, polyunsaturated fatty acids; S, small; SFA, saturated fatty acids; VLDL, very low-density lipoproteins; XL, very large; XS, very small; XXL, extremely large

**Fig. 3:**
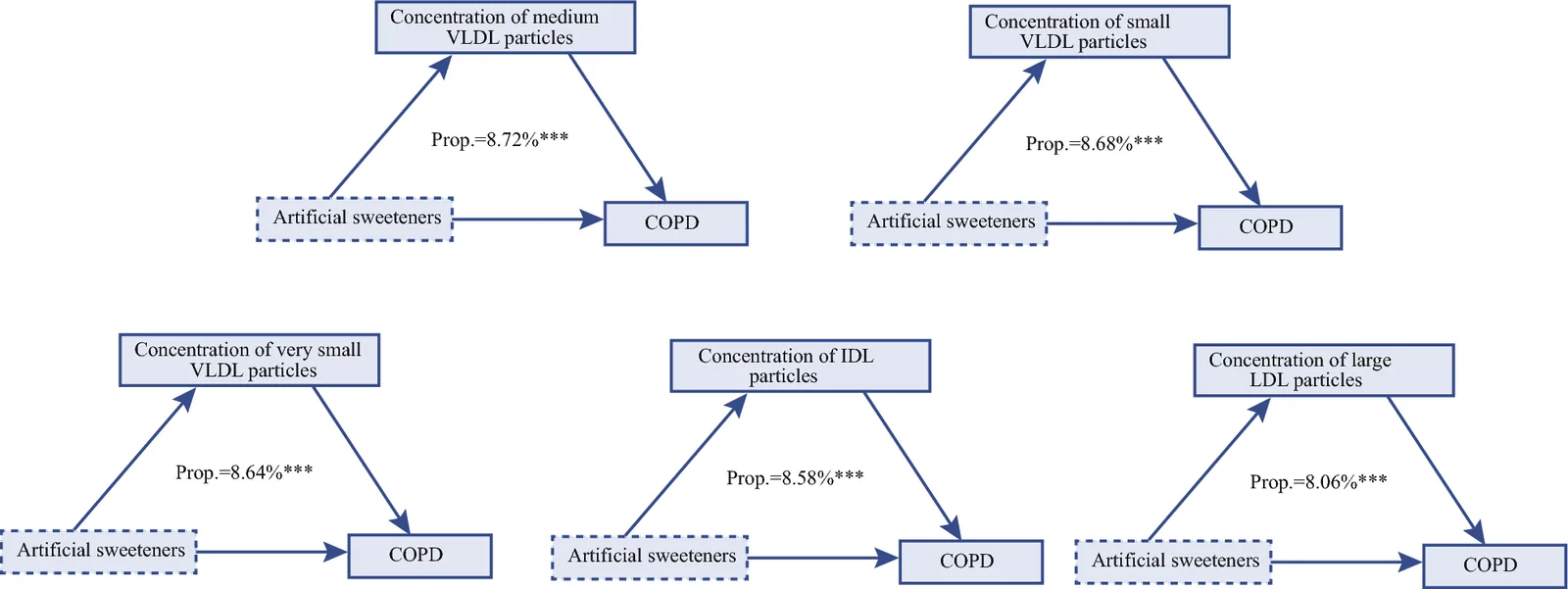
Mediating effects of key mediating metabolic biomarkers on the associations between artificial sweetener intake and COPD risk: cholesterol in IDL, total lipids in large LDL, free cholesterol in IDL, cholesteryl esters in IDL, phospholipids in large LDL. Models were adjusted for age, sex, ethnicity, educational attainmen, socioeconomic deprivation index, body mass index, smoking status, alcohol drinking, healthy diet, leisure time physical activity, family history of lung diseases, diabetes, and air pollutants (NO_2_, NO_X_, PM_2.5_, PM_10_, PM_2.5–10_). Prop., proportion mediated; ***P < 0.001

We performed a comprehensive mediation analysis to assess the role of 143 circulating metabolites in the association between artificial sweetener consumption and COPD. After FDR correction for testing 143 metabolites, 63 showed significant mediating effects. Lipoproteins constituted the majority of findings (n = 50; 79.4% of selected metabolites), involved key components such as cholesterol, cholesteryl esters, and phospholipids. Additionally, we identified significant fatty acids (n = 8), as well as other lipids (n = 4) and other metabolites (n = 1) (Fig. 2, Supplementary Table S6). The most prominent mediators were components of low-density lipoprotein (LDL) and related particles (Fig. 3). Phospholipids in large LDL showed the highest mediating effect (PM: 8.72%, 95% CI: 4.53% to 19.63%). Similarly, the cholesteryl esters content in intermediate-density lipoprotein (IDL) demonstrated significant mediation (PM: 8.68%, 95% CI: 4.78% to 20.58%), as did cholesterol in large, medium, and small LDL particles (PM: 4.20%, 5.86%, and 5.80%, respectively). Beyond lipoprotein subfractions, apolipoprotein B (ApoB) served as a significant mediator (PM: 6.70%, 95% CI: 3.67% to 16.35%), while the ApoB to ApoA1 ratio exhibited a weaker effect (PM: 1.87%, 95% CI: 0.53% to 4.99%). Furthermore, key fatty acids were identified as significant mediators, including linoleic acid (PM: 7.16%, 95% CI: 3.92% to 16.47%) and the total pool of polyunsaturated fatty acids (PM: 6.55%, 95% CI: 3.35% to 18.14%). In conclusion, the relationship between artificial sweetener intake and COPD is partially mediated through multiple pathways related to LDL metabolism, ApoB, and specific fatty acids, with each explaining a small portion of the total effect.

## Discussion

This large-scale prospective cohort study within the UK Biobank demonstrated that participants with higher consumption of artificial sweeteners had an elevated risk of COPD and a decreased lung function level compared to those without consumption of artificial sweeteners. These associations remained robust after extensive adjustment for potential confounders and were consistent across all sensitivity analyses. Our mediation analysis indicates that the association between artificial sweetener consumption and COPD is partially explained by the concerted action of specific metabolites. These metabolites are primarily involved in LDL metabolism, apolipoprotein B, and key polyunsaturated fatty acids. However, the proportion of the total effect mediated by each individual pathway was modest.

The potential relationship between artificial sweetener intake and COPD risk remains an unexamined area in nutritional epidemiology. While previous research has explored the role of diet in chronic respiratory diseases, investigations into UPFs and COPD have yielded conflicting conclusions [12, 31]. This inconsistency may stem from the substantial heterogeneity inherent in the UPF category, which encompasses a wide array of products with divergent nutritional compositions, thereby obscuring the specific effects of constituents like artificial sweeteners. More direct evidence comes from studies on sugary beverages. A recent large-scale prospective cohort study from the UK Biobank reported that higher consumption of ASBs was associated with an increased risk of incident COPD and asthma [13]. Similarly, several cross-sectional studies have identified a positive association between ASBs consumption and the prevalence of both COPD and asthma [32–36]. These observations direct attention to the common feature of beverage sweeteners. However, much of the existing literature has focused on the detrimental role of sugars in sugar-sweetened beverages or has used ASBs as a proxy for artificial sweeteners. This approach fails to isolate the independent effect of artificial sweeteners themselves, as the observed associations could be confounded by other compounds present in these beverages. Furthermore, a significant limitation of prior studies is the general neglect of tabletop artificial sweeteners (e.g., those added to coffee, tea, or food), which constitute a major dietary source of artificial sweeteners and have simpler ingredient lists, potentially providing a clearer perspective on their health effects. Our study was specifically designed to address this pivotal question.

To generate hypotheses on potential biological pathways, we conducted an exploratory mediation analysis to identify metabolites that may mediate the association between artificial sweetener intake and COPD risk. After FDR correction for testing 143 metabolites, 63 showed significant mediating effects. The significant mediators were predominantly clustered within a few highly correlated metabolic classes, notably pro-atherogenic lipoprotein profiles (e.g., medium-density and all subclasses of LDL particles and apolipoprotein B) and polyunsaturated fatty acids (e.g., linoleic acid). However, it is crucial to emphasize that the mediating proportions were modest (< 10%) and that significant col-linearity exists among these metabolites. Therefore, these results should be interpreted with caution and are primarily hypothesis-generating rather than conclusive proof of mechanism. These findings are consistent with previous research linking lipid metabolism to respiratory health. A recent large-scale study using UK Biobank data investigated the association between air pollution exposure and COPD risk [24]. The study found that air pollution was associated with both lung function decline and increased COPD incidence. Importantly, mediation analysis revealed that lipid-related metabolites significantly mediated this relationship [24]. Previous studies have suggested that the lungs represent a target organ of systemic damage in cardiovascular disease (CVD) [37]. Existing CVD conditions, such as heart failure and ischemic heart disease, have been shown to significantly increase the risk of disease exacerbation and mortality in COPD patients. Arterial stiffness, a modifiable risk factor for CVD, is markedly elevated in individuals with COPD, indicating its potential utility in risk stratification and as a target for intervention [38, 39]. Nonetheless, these exploratory findings offer novel, data-driven clues for generating hypotheses about a potential link between artificial sweetener intake and respiratory health. Future research is needed to specifically test in more controlled settings (e.g., interventional trials or animal models) whether and how these specific metabolic pathways mediate the effects of artificial sweeteners on lung function.

Our metabolomic analysis points to systemic inflammatory and oxidative stress pathways, which are established hallmarks of COPD. While observational data cannot establish direct biological mechanisms, findings from experimental models offer a plausible framework to interpret these associations. The biological plausibility of this link is supported by two convergent lines of evidence. First, a hallmark of COPD itself is a state of systemic inflammation, characterized by elevated circulating levels of cytokines such as CRP, IL-6, and TNF-α [40, 41]. Second, and independently, experimental studies suggest that artificial sweeteners may promote a similar pro-inflammatory and pro-oxidative state. For instance, studies in animals and in vitro systems suggest that commonly used artificial sweeteners like aspartame and sucralose can induce the production of key pro-inflammatory cytokines (e.g., TNF-α, IL-6) and oxidative stress [42–45]. At the same time, chronic consumption of artificial sweeteners alters the gut microbiota by reducing its diversity and enriching pro-inflammatory genes [46, 47]. These changes together create a self-reinforcing cycle of systemic inflammation. These alterations extend beyond the gut, correlating with histopathological damage and elevated inflammatory markers in the liver and other organs [48, 49], thereby establishing a pervasive pro-inflammatory milieu. Evidence for acesulfame-K and saccharin is less consistent, but studies in animals show that Ace-K can trigger gut inflammation and increase intestinal permeability [50, 51]. It is crucial to emphasize that these mechanistic insights are derived primarily from controlled, often high-dose, experimental settings. The extent to which these pathways are activated and contribute to disease in humans following chronic, low-level consumption of artificial sweeteners remains to be fully elucidated and represents a key area for future research. While acknowledging the limitations of cross-study comparisons, the convergence of our epidemiological findings with these independent experimental observations may strengthen the biological plausibility of the association we report.

In addition to analyzing the overall association between artificial sweetener consumption and COPD, we also conducted subgroup analyses based on socioeconomic status, familial factors, and environmental exposures, as identifying susceptible sub-populations is crucial for developing targeted strategies to mitigate the disease burden. Our subgroup analysis revealed that smoking status significantly modified the association between artificial sweetener consumption and COPD risk, suggesting a potential biological interaction between these factors. Cigarette smoke imposes a substantial oxidant burden and provokes a persistent inflammatory state in the lungs, potentially rendering them more vulnerable to secondary insults [52, 53]. Smokers tend to exhibit other unhealthy lifestyle behaviors, such as higher intake of sugars and processed foods. Therefore, the elevated risk observed in the smoking population may partially result from the synergistic effects of “unhealthy dietary patterns” and “smoking.” In disadvantaged populations, smoking often coexists with other adverse factors including alcohol drinking, poor dietary habits and others, forming clustered behavioral risk factors that collectively increase the cumulative risk of comorbidities [54]. The consumption of artificial sweeteners may merely serve as a group of such unhealthy dietary patterns. Despite statistical adjustments for known confounders, residual confounding remains a concern. The influence of the overall dietary pattern is difficult to capture precisely, and this effect may be particularly relevant among smokers.

This study has several strengths. First, the prospective design and the large sample size from the UK Biobank provide substantial statistical power for our analyses. The cohort’s detailed baseline characterization, encompassing socioeconomic status, dietary factors, lifestyle, environmental exposures, and medical history, allowed for comprehensive adjustment for a wide array of potential confounders. Second, our study is the first to provide an in-depth examination of the relationships between artificial sweetener intake and COPD incidence and lung function, including a comprehensive analysis of variability across key subgroups. Third, our work breaks new ground by moving beyond established risk factors to interrogate the role of the plasma metabolome. The formal mediation analysis represents a major analytical step, generating preliminary hypotheses regarding specific metabolic shifts that might link sweetener intake to respiratory outcomes. Finally, the consistency of our primary findings across multiple sensitivity checks reinforces the reliability of the observed relationships.

Several limitations must be acknowledged. First, due to the observational nature of this study, a causal relationship cannot be established. Artificial sweetener use clusters with obesity, smoking, diabetes, and lower socioeconomic status. Although we adjusted for these factors, it remains difficult to distinguish a direct causal effect from behavioral marking of an underlying high-risk profile, a concern underscored by stronger associations in smokers and inactive participants. Moreover, reverse causation is possible, as individuals with obesity or metabolic disturbances may selectively consume artificial sweeteners as a sugar substitute, potentially introducing indication bias, which may partly explain the attenuated association in the obese subgroup. To mitigate this concern, sensitivity analyses excluding short follow-up periods (3 and 5 years) yielded consistent results. Nonetheless, residual reverse causality cannot be entirely ruled out. Second, our COPD ascertainment, based on hospital and mortality codes, likely captures more severe disease and may lack specificity without post-bronchodilator spirometry. This limits inferences regarding early disease processes. Third, the mediation analysis is exploratory. The simultaneous testing of numerous, highly correlated metabolites raises concerns regarding multiple testing and collinearity, and the small mediated proportions preclude strong causal inference. These results are hypothesis-generating and require experimental validation. Fourth, measurement bias may exist. Artificial sweetener intake was assessed by self-reported dietary data, which may not be fully accurate. Additionally, selection bias may arise from the exclusion of participants without complete dietary data, potentially limiting generalizability. Compared with the general UK population, UK Biobank participants are more likely to be older, female, and live in more socio-economically advantaged areas, and to be healthier [55]. Therefore, our findings may represent conservative estimates, and generalization to the total UK population and other populations cannot be made. Additional validation through clinical trials or intervention studies is warranted before the results can be applied practically. Finally, COPD diagnosis in the UK Biobank was based on self-report and hospital records, without spirometry confirmation. This may introduce outcome misclassification, particularly for mild or early-stage cases. We were also unable to examine individual sweetener subtypes.

## Conclusions

In this population-based large prospective cohort, the results demonstrated that higher consumption of artificial sweeteners is associated with an increased risk of incident COPD and a decline lung function level, with a significant dose–response relationship observed. This association is partially mediated by specific circulating metabolites, particularly those low-and intermediate-density lipoproteins metabolism. The observed relationships were robust across multiple sensitivity analyses. These findings highlight the potential adverse respiratory effects of artificial sweeteners and suggest that reducing their consumption may contribute to the prevention of COPD. Further experimental studies are warranted to elucidate the underlying biological mechanisms and confirm the causal nature of these associations.

## Supplementary Information

Additional file 1.

## Data Availability

The datasets analyzed during the current study are available in a public, open-access repository (https://www.ukbiobank.ac.uk). All data used in this study were obtained from the UK Biobank under Application Number 1126547.
