# Supplementary material for: Artificial sweetener intake and risk of chronic obstructive pulmonary disease: a mediation analysis based on plasma metabolic biomarkers
Source: Nutr Metab (Lond). 2026 Jul 7;23:115. doi: 10.1186/s12986-026-01142-z (PMC13637212; doi:10.1186/s12986-026-01142-z)
Supplement: Supplementary file 1 [file 12986_2026_1142_MOESM1_ESM.docx]

**Supporting Information**

**Supporting Information Methods**

**Supplementary Figure 1.** Study design and participant selection flowchart.

**Supplementary Table S1.** Subgroup of the association between artificial sweetener intake and incident COPD risk

**Supplementary Table S2.** Subgroup of the association between artificial sweetener intake and FVC level.

**Supplementary Table S3.** Subgroup of the association between artificial sweetener intake and FEV_1_ level.

**Supplementary Table S4.** Sensitivity analysis of the association between artificial sweetener intake and incident COPD risk, excluding participants with follow-up duration shorter than 3 or 5 years.

**Supplementary Table S5.** Subgroup of the association between artificial sweetener intake and incident COPD risk, excluding participants with follow-up duration shorter than 3 years.

**Supplementary Table S6.** The estimated mediation proportions for identified metabolites on the associations of artificial sweeteners with COPD.

**Supporting Information Methods**

**Study population**

During their initial assessment, subjects finished a digital self-reported survey and received a series of physical examinations. A wide range of information regarding personal exposures and health conditions was gathered at enrollment; concurrently, biospecimens were donated for subsequent laboratory analyses. Electronic informed consent was secured from every participant. Ethical clearance for the UK Biobank was granted by relevant regulatory bodies, including the North West Multi-centre Research Ethics Committee and corresponding national governance entities in England, Wales, and Scotland ([http://www.ukbiobank.ac.uk/ethics/](http://www.ukbiobank.ac.uk/ethics/)).

This study utilized data from 501,950 participants in the UK Biobank. Initially, 291,206 individuals who did not complete at least one valid 24-hour dietary recall questionnaire were excluded, resulting in a lack of detailed dietary intake information, including data on artificial sweetener consumption. Subsequently, we excluded an additional 43,545 participants with “low-calorie drink intake”, as such beverages often contain multiple additives, making it difficult to accurately quantify specific artificial sweetener intake. Furthermore, 368 individuals with “varied” artificial sweetener intake and 2,175 participants diagnosed with COPD prior to baseline assessment were also excluded[23]. Ultimately, 164,656 participants were included in the study. For the analysis of lung function, an additional 42,231 participants lacking pulmonary function measurements were further excluded.

**Supplementary Figure 1.** Study design and participant selection flowchart.

**Supplementary Table S1.** Subgroup of the association between artificial sweetener intake and incident COPD risk.

| Variables | Subgroups | Per 1-teaspoon increase  In artificial sweeteners | | Non-consumers | Lower consumers | | Higher consumers | | P for  interaction |
| --- | --- | --- | --- | --- | --- | --- | --- | --- | --- |
|  |  | HR (95% CI) | P-value | HR (95% CI) | HR (95% CI) | P-value | HR (95% CI) | P-value |  |
| Age | <60 years | 1.01 (0.99, 1.03) | 0.163 | 1.0 (Ref.) | 1.11 (0.88, 1.39) | 0.397 | 1.13 (0.92, 1.39) | 0.231 | 0.480 |
|  | ≥60 years | 1.03 (1.02, 1.04) | <0.001 | 1.0 (Ref.) | 1.09 (0.95, 1.26) | 0.218 | 1.35 (1.20, 1.52) | <0.001 |  |
| Sex | Male | 1.03 (1.01, 1.04) | <0.001 | 1.0 (Ref.) | 1.04 (0.88, 1.24) | 0.623 | 1.29 (1.13, 1.47) | <0.001 | 0.605 |
|  | Female | 1.02 (1.00, 1.04) | 0.011 | 1.0 (Ref.) | 1.12 (0.95, 1.33) | 0.188 | 1.21 (1.03, 1.42) | 0.023 |  |
| Educational  attainment | College or  University degree | 1.03 (1.01, 1.06) | 0.003 | 1.0 (Ref.) | 1.49 (1.17, 1.90) | 0.001 | 1.33 (1.04, 1.69) | 0.022 | 0.393 |
|  | Others | 1.02 (1.01, 1.03) | <0.001 | 1.0 (Ref.) | 0.99 (0.86, 1.14) | 0.895 | 1.25 (1.11, 1.40) | <0.001 |  |
| Socioeconomic  deprivation index | Low | 1.03 (1.01, 1.04) | <0.001 | 1.0 (Ref.) | 1.00 (0.82, 1.22) | 0.974 | 1.35 (1.15, 1.58) | <0.001 | 0.728 |
|  | High | 1.02 (1.01, 1.04) | <0.001 | 1.0 (Ref.) | 1.13 (0.97, 1.32) | 0.131 | 1.21 (1.06, 1.39) | 0.006 |  |
| Body mass index | <30 kg/m^2^ | 1.03 (1.02, 1.04) | <0.001 | 1.0 (Ref.) | 1.03 (0.89, 1.21) | 0.663 | 1.31 (1.16, 1.49) | <0.001 | 0.089 |
|  | ≥30 kg/m^2^ | 1.01 (1.00, 1.03) | 0.135 | 1.0 (Ref.) | 1.09 (0.89, 1.34) | 0.380 | 1.16 (0.97, 1.39) | 0.094 |  |
| Smoking status | Never | 0.99 (0.96, 1.03) | 0.710 | 1.0 (Ref.) | 1.27 (0.98, 1.64) | 0.067 | 0.90 (0.65, 1.25) | 0.536 | 0.010 |
|  | Previous or current | 1.04 (1.03, 1.05) | <0.001 | 1.0 (Ref.) | 1.01 (0.88, 1.16) | 0.855 | 1.42 (1.27, 1.59) | <0.001 |  |
| Alcohol drinking | Never or previous | 1.02 (1.00, 1.05) | 0.101 | 1.0 (Ref.) | 0.97 (0.61, 1.54) | 0.885 | 1.09 (0.80, 1.50) | 0.582 | 0.780 |
|  | Current | 1.02 (1.01, 1.04) | <0.001 | 1.0 (Ref.) | 1.08 (0.95, 1.23) | 0.230 | 1.28 (1.15, 1.43) | <0.001 |  |
| Healthy diet | No | 1.02 (1.01, 1.03) | 0.003 | 1.0 (Ref.) | 1.03 (0.90, 1.18) | 0.643 | 1.18 (1.05, 1.33) | 0.005 | 0.785 |
|  | Yes | 1.05 (1.03, 1.08) | <0.001 | 1.0 (Ref.) | 1.40 (1.03, 1.89) | 0.031 | 1.81 (1.38, 2.35) | <0.001 |  |
| Leisure time  physical activity | <1000 MET mins/week | 1.03 (1.01, 1.04) | <0.001 | 1.0 (Ref.) | 0.99 (0.84, 1.17) | 0.909 | 1.26 (1.10, 1.44) | <0.001 | 0.018 |
|  | ≥1000 MET mins/week | 1.03 (1.01, 1.05) | 0.001 | 1.0 (Ref.) | 1.22 (0.99, 1.51) | 0.063 | 1.32 (1.09, 1.60) | 0.004 |  |
| Diabetes | No | 1.02 (1.01, 1.03) | <0.001 | 1.0 (Ref.) | 1.05 (0.92, 1.20) | 0.474 | 1.24 (1.11, 1.39) | <0.001 | 0.126 |
|  | Yes | 1.05 (1.02, 1.07) | <0.001 | 1.0 (Ref.) | 1.33 (0.95, 1.86) | 0.101 | 1.57 (1.18, 2.08) | 0.002 |  |
| Family history of  chronic lung disease | No | 1.02 (1.01, 1.03) | <0.001 | 1.0 (Ref.) | 1.11 (0.97, 1.28) | 0.137 | 1.25 (1.11, 1.41) | <0.001 | 0.982 |
|  | Yes | 1.03 (1.01, 1.05) | 0.006 | 1.0 (Ref.) | 1.02 (0.79, 1.31) | 0.907 | 1.30 (1.05, 1.60) | 0.014 |  |
| NO_2_ | Low | 1.02 (1.01, 1.04) | 0.003 | 1.0 (Ref.) | 1.02 (0.85, 1.22) | 0.872 | 1.27 (1.09, 1.47) | 0.002 | 0.847 |
|  | High | 1.02 (1.01, 1.04) | <0.001 | 1.0 (Ref.) | 1.15 (0.97, 1.36) | 0.100 | 1.22 (1.06, 1.42) | 0.007 |  |
| NO_X_ | Low | 1.03 (1.01, 1.04) | <0.001 | 1.0 (Ref.) | 1.05 (0.87, 1.26) | 0.615 | 1.30 (1.11, 1.51) | <0.001 | 0.452 |
|  | High | 1.02 (1.01, 1.03) | 0.004 | 1.0 (Ref.) | 1.12 (0.94, 1.32) | 0.194 | 1.20 (1.04, 1.38) | 0.015 |  |
| PM_2.5_ | Low | 1.03 (1.02, 1.05) | <0.001 | 1.0 (Ref.) | 1.15 (0.96, 1.38) | 0.130 | 1.36 (1.16, 1.59) | <0.001 | 0.128 |
|  | High | 1.02 (1.00, 1.03) | 0.028 | 1.0 (Ref.) | 1.02 (0.85, 1.21) | 0.846 | 1.16 (1.00, 1.35) | 0.050 |  |
| PM_10_ | Low | 1.02 (1.01, 1.04) | 0.004 | 1.0 (Ref.) | 1.12 (0.94, 1.34) | 0.194 | 1.21 (1.03, 1.41) | 0.017 | 0.836 |
|  | High | 1.02 (1.01, 1.04) | <0.001 | 1.0 (Ref.) | 1.03 (0.87, 1.23) | 0.718 | 1.27 (1.10, 1.47) | 0.001 |  |
| PM_2.5-10_ | Low | 1.03 (1.02, 1.05) | <0.001 | 1.0 (Ref.) | 1.09 (0.91, 1.31) | 0.330 | 1.27 (1.09, 1.49) | 0.002 | 0.196 |
|  | High | 1.02 (1.00, 1.03) | 0.035 | 1.0 (Ref.) | 1.06 (0.89, 1.26) | 0.499 | 1.22 (1.05, 1.41) | 0.009 |  |

Abbreviations: NO_2_, nitrogen dioxide; NO_X_, nitrogen oxides; PM_2.5_, particulate matter with a diameter ≤2.5 μm; PM_10_, particulate matter with a diameter ≤10 μm

Model was adjusted age, sex, ethnicity (White, Mixed, South Asian, Black, Chinese, other races, or missing), educational attainment (no relevant qualifications, college or university degree, A levels/AS levels or equivalent, O levels/GCSEs or equivalent, CSEs or equivalent, others, or missing), socioeconomic deprivation index (quintiles, or missing), body mass index (<18.5, 18.5 to <25, 25 to <30, ≥30 kg/m^2,^ or missing), smoking status (never, previous, current, or missing), alcohol drinking (never, previous, current, or missing), healthy diet (yes, no, or missing), leisure time physical activity (<500, 500 to <1000, ≥1000 MET mins/week, or missing), family history of lung diseases (yes, no, or missing), diabetes (yes, no, or missing), air pollutants (NO_2_ [quintiles, or missing], NO_X_ [quintiles, or missing], PM_2.5_ [quintiles, or missing], PM_10_ [quintiles, or missing], PM_2.5-10_ [quintiles, or missing]).

**Supplementary Table S2.** Subgroup of the association between artificial sweetener intake and FVC level.

| Variables | Subgroups | Per 1-teaspoon increase  In artificial sweeteners | | Non-consumers | Lower consumers | | Higher consumers | | P for  interaction |
| --- | --- | --- | --- | --- | --- | --- | --- | --- | --- |
|  |  | β (95% CI) | P-value | β (95% CI) | β (95% CI) | P-value | β (95% CI) | P-value |  |
| Age | <60 years | -4.79 (-7.46, -2.13) | <0.001 | 0.0 (Ref.) | -26.46 (-51.35, -1.57) | 0.037 | -47.44 (-73.28, -21.61) | <0.001 | 0.613 |
|  | ≥60 years | -4.21 (-6.66, -1.77) | <0.001 | 0.0 (Ref.) | -36.23 (-59.35, -13.11) | 0.002 | -50.19 (-73.43, -26.95) | <0.001 |  |
| Sex | Male | -5.05 (-7.90, -2.20) | <0.001 | 0.0 (Ref.) | -44.65 (-74.24, -15.06) | 0.003 | -55.95 (-83.93, -27.98) | <0.001 | 0.025 |
|  | Female | -1.05 (-3.18, 1.09) | 0.336 | 0.0 (Ref.) | -14.94 (-33.22, 3.35) | 0.109 | -13.23 (-33.22, 6.77) | 0.195 |  |
| Educational  attainment | College or  University degree | -5.22 (-8.67, -1.78) | 0.003 | 0.0 (Ref.) | -43.86 (-73.47, -14.25) | 0.004 | -48.04 (-80.21, -15.87) | 0.003 | 0.379 |
|  | Others | -3.42 (-5.49, -1.34) | 0.001 | 0.0 (Ref.) | -23.95 (-44.19, -3.71) | 0.02 | -38.32 (-58.43, -18.20) | <0.001 |  |
| Socioeconomic  deprivation index | Low | -2.30 (-4.85, 0.25) | 0.077 | 0.0 (Ref.) | -36.39 (-59.39, -13.39) | 0.002 | -38.08 (-62.05, -14.11) | 0.002 | 0.124 |
|  | High | -5.09 (-7.57, -2.61) | <0.001 | 0.0 (Ref.) | -19.01 (-43.35, 5.32) | 0.126 | -41.06 (-65.35, -16.77) | <0.001 |  |
| Body mass index | <30 kg/m^2^ | -6.08 (-8.23, -3.94) | <0.001 | 0.0 (Ref.) | -43.46 (-63.02, -23.90) | <0.001 | -56.52 (-76.93, -36.11) | <0.001 | 0.029 |
|  | ≥30 kg/m^2^ | -1.48 (-4.76, 1.80) | 0.376 | 0.0 (Ref.) | -25.72 (-59.01, 7.58) | 0.130 | -30.65 (-62.86, 1.57) | 0.062 |  |
| Smoking status | Never | -2.99 (-5.88, -0.11) | 0.042 | 0.0 (Ref.) | -30.58 (-54.20, -6.95) | 0.011 | -19.70 (-46.23, 6.82) | 0.145 | 0.446 |
|  | Previous or current | -4.28 (-6.54, -2.01) | <0.001 | 0.0 (Ref.) | -24.14 (-47.83, -0.45) | 0.046 | -55.06 (-77.43, -32.69) | <0.001 |  |
| Alcohol drinking | Never or previous | -3.16 (-9.00, 2.67) | 0.288 | 0.0 (Ref.) | -11.50 (-80.98, 57.98) | 0.746 | -53.22 (-115.48, 9.03) | 0.094 | 0.869 |
|  | Current | -3.72 (-5.58, -1.85) | <0.001 | 0.0 (Ref.) | -29.20 (-46.41, -12.00) | <0.001 | -38.20 (-55.93, -20.48) | <0.001 |  |
| Healthy diet | No | -3.29 (-5.25, -1.33) | 0.001 | 0.0 (Ref.) | -31.06 (-49.50, -12.61) | 0.001 | -37.99 (-56.71, -19.28) | <0.001 | 0.679 |
|  | Yes | -4.37 (-9.07, 0.32) | 0.068 | 0.0 (Ref.) | -12.64 (-54.05, 28.78) | 0.550 | -43.94 (-89.09, 1.22) | 0.057 |  |
| Leisure time  physical activity | <1000 MET  mins/week | -3.56 (-5.93, -1.20) | 0.003 | 0.0 (Ref.) | -26.54 (-48.43, -4.65) | 0.018 | -40.55 (-63.03, -18.08) | <0.001 | 0.137 |
|  | ≥1000 MET  mins/week | -5.47 (-8.42, -2.52) | <0.001 | 0.0 (Ref.) | -33.68 (-61.13, -6.23) | 0.016 | -54.88 (-83.19, -26.56) | <0.001 |  |
| Diabetes | No | -4.06 (-5.92, -2.20) | <0.001 | 0.0 (Ref.) | -29.42 (-46.69, -12.16) | <0.001 | -41.01 (-58.75, -23.27) | <0.001 | 0.039 |
|  | Yes | 2.55 (-3.73, 8.83) | 0.427 | 0.0 (Ref.) | 5.90 (-60.54, 72.35) | 0.862 | 3.05 (-60.78, 66.88) | 0.925 |  |
| Family history of  chronic lung disease | No | -4.39 (-6.36, -2.42) | <0.001 | 0.0 (Ref.) | -26.97 (-45.35, -8.60) | 0.004 | -45.67 (-64.57, -26.77) | <0.001 | 0.140 |
|  | Yes | -0.78 (-5.06, 3.50) | 0.722 | 0.0 (Ref.) | -36.13 (-77.85, 5.59) | 0.090 | -15.60 (-56.90, 25.69) | 0.459 |  |
| NO_2_ | Low | -3.12 (-5.62, -0.61) | 0.015 | 0.0 (Ref.) | -26.62 (-49.69, -3.56) | 0.024 | -38.90 (-62.58, -15.22) | 0.001 | 0.333 |
|  | High | -4.53 (-7.14, -1.91) | <0.001 | 0.0 (Ref.) | -29.44 (-54.48, -4.39) | 0.021 | -43.63 (-69.07, -18.19) | <0.001 |  |
| NO_X_ | Low | -2.90 (-5.44, -0.36) | 0.025 | 0.0 (Ref.) | -29.50 (-52.81, -6.18) | 0.013 | -37.42 (-61.56, -13.28) | 0.002 | 0.247 |
|  | High | -4.62 (-7.20, -2.05) | <0.001 | 0.0 (Ref.) | -26.39 (-51.13, -1.65) | 0.037 | -44.46 (-69.38, -19.54) | <0.001 |  |
| PM_2.5_ | Low | -2.32 (-4.97, 0.34) | 0.087 | 0.0 (Ref.) | -30.81 (-55.12, -6.51) | 0.013 | -33.15 (-58.02, -8.27) | 0.009 | 0.150 |
|  | High | -4.95 (-7.55, -2.36) | <0.001 | 0.0 (Ref.) | -28.29 (-53.15, -3.43) | 0.026 | -45.49 (-70.87, -20.11) | <0.001 |  |
| PM_10_ | Low | -3.37 (-5.98, -0.75) | 0.012 | 0.0 (Ref.) | -24.46 (-48.69, -0.23) | 0.048 | -38.09 (-62.92, -13.26) | 0.003 | 0.618 |
|  | High | -3.96 (-6.59, -1.33) | 0.003 | 0.0 (Ref.) | -35.50 (-60.45, -10.55) | 0.005 | -39.72 (-65.16, -14.28) | 0.002 |  |
| PM_2.5-10_ | Low | -3.80 (-6.42, -1.17) | 0.005 | 0.0 (Ref.) | -29.02 (-53.41, -4.64) | 0.020 | -38.62 (-63.87, -13.37) | 0.003 | 0.840 |
|  | High | -3.55 (-6.17, -0.93) | 0.008 | 0.0 (Ref.) | -31.10 (-55.87, -6.33) | 0.014 | -38.93 (-63.92, -13.93) | 0.002 |  |

Abbreviations: NO_2_, nitrogen dioxide; NO_X_, nitrogen oxides; PM_2.5_, particulate matter with a diameter ≤2.5 μm; PM_10_, particulate matter with a diameter ≤10 μm

Model was adjusted age, sex, ethnicity (White, Mixed, South Asian, Black, Chinese, other races, or missing), educational attainment (no relevant qualifications, college or university degree, A levels/AS levels or equivalent, O levels/GCSEs or equivalent, CSEs or equivalent, others, or missing), socioeconomic deprivation index (quintiles, or missing), body mass index (<18.5, 18.5 to <25, 25 to <30, ≥30 kg/m^2,^ or missing), smoking status (never, previous, current, or missing), alcohol drinking (never, previous, current, or missing), healthy diet (yes, no, or missing), leisure time physical activity (<500, 500 to <1000, ≥1000 MET mins/week, or missing), family history of lung diseases (yes, no, or missing), diabetes (yes, no, or missing), air pollutants (NO_2_ [quintiles, or missing], NO_X_ [quintiles, or missing], PM_2.5_ [quintiles, or missing], PM_10_ [quintiles, or missing], PM_2.5-10_ [quintiles, or missing]).

**Supplementary Table S3.** Subgroup of the association between artificial sweetener intake and FEV_1_ level.

| Variables | Subgroups | Per 1-teaspoon increase  In artificial sweeteners | | Non-consumers | Lower consumers | | Higher consumers | | P for  interaction |
| --- | --- | --- | --- | --- | --- | --- | --- | --- | --- |
|  |  | β (95% CI) | P-value | β (95% CI) | β (95% CI) | P-value | β (95% CI) | P-value |  |
| Age | <60 years | -4.21 (-6.39, -2.04) | <0.001 | 0.0 (Ref.) | -15.95 (-36.29, 4.39) | 0.124 | -40.44 (-61.55, -19.33) | <0.001 | 0.991 |
|  | ≥60 years | -4.70 (-6.71, -2.70) | <0.001 | 0.0 (Ref.) | -26.91 (-45.86, -7.97) | 0.005 | -51.47 (-70.51, -32.43) | <0.001 |  |
| Sex | Male | -4.72 (-7.04, -2.40) | <0.001 | 0.0 (Ref.) | -32.69 (-56.76, -8.62) | 0.008 | -50.11 (-72.86, -27.35) | <0.001 | 0.038 |
|  | Female | -1.69 (-3.42, 0.03) | 0.055 | 0.0 (Ref.) | -5.96 (-20.73, 8.81) | 0.429 | -17.02 (-33.17, -0.87) | 0.039 |  |
| Educational  attainment | College or  University degree | -4.44 (-7.23, -1.64) | 0.002 | 0.0 (Ref.) | -31.70 (-55.72, -7.68) | 0.01 | -41.94 (-68.03, -15.84) | 0.002 | 0.615 |
|  | Others | -3.64 (-5.32, -1.95) | <0.001 | 0.0 (Ref.) | -14.21 (-30.62, 2.20) | 0.09 | -37.18 (-53.49, -20.87) | <0.001 |  |
| Socioeconomic  deprivation index | Low | -2.94 (-5.01, -0.88) | 0.005 | 0.0 (Ref.) | -21.43 (-40.03, -2.84) | 0.024 | -39.89 (-59.26, -20.52) | <0.001 | 0.241 |
|  | High | -4.44 (-6.46, -2.42) | <0.001 | 0.0 (Ref.) | -14.83 (-34.64, 4.98) | 0.142 | -34.27 (-54.05, -14.49) | <0.001 |  |
| Body mass index | <30 kg/m^2^ | -4.87 (-6.60, -3.14) | <0.001 | 0.0 (Ref.) | -21.54 (-37.28, -5.80) | 0.007 | -45.64 (-62.07, -29.22) | <0.001 | 0.124 |
|  | ≥30 kg/m^2^ | -2.27 (-4.99, 0.45) | 0.102 | 0.0 (Ref.) | -21.52 (-49.15, 6.10) | 0.127 | -29.63 (-56.35, -2.90) | 0.030 |  |
| Smoking status | Never | -1.71 (-4.02, 0.60) | 0.147 | 0.0 (Ref.) | -17.31 (-36.26, 1.63) | 0.073 | -7.21 (-28.48, 14.07) | 0.507 | 0.010 |
|  | Previous or current | -5.33 (-7.20, -3.46) | <0.001 | 0.0 (Ref.) | -16.90 (-36.44, 2.64) | 0.090 | -62.85 (-81.30, -44.41) | <0.001 |  |
| Alcohol drinking | Never or previous | -3.73 (-8.44, 0.98) | 0.121 | 0.0 (Ref.) | -12.23 (-68.32, 43.86) | 0.669 | -47.31 (-97.57, 2.95) | 0.065 | 0.908 |
|  | Current | -3.63 (-5.14, -2.11) | <0.001 | 0.0 (Ref.) | -18.85 (-32.81, -4.89) | 0.008 | -35.92 (-50.30, -21.54) | <0.001 |  |
| Healthy diet | No | -3.26 (-4.85, -1.67) | <0.001 | 0.0 (Ref.) | -19.39 (-34.35, -4.43) | 0.011 | -35.30 (-50.48, -20.12) | <0.001 | 0.604 |
|  | Yes | -4.37 (-8.18, -0.56) | 0.025 | 0.0 (Ref.) | -10.99 (-44.60, 22.63) | 0.522 | -40.25 (-76.90, -3.59) | 0.031 |  |
| Leisure time  physical activity | <1000 MET  mins/week | -4.41 (-6.33, -2.48) | <0.001 | 0.0 (Ref.) | -15.96 (-33.74, 1.82) | 0.079 | -46.11 (-64.37, -27.85) | <0.001 | 0.690 |
|  | ≥1000 MET  mins/week | -4.32 (-6.70, -1.94) | <0.001 | 0.0 (Ref.) | -25.40 (-47.59, -3.20) | 0.025 | -40.60 (-63.49, -17.70) | <0.001 |  |
| Diabetes | No | -4.02 (-5.52, -2.51) | <0.001 | 0.0 (Ref.) | -18.47 (-32.47, -4.46) | 0.010 | -37.98 (-52.36, -23.59) | <0.001 | 0.051 |
|  | Yes | 1.60 (-3.55, 6.75) | 0.542 | 0.0 (Ref.) | -6.70 (-61.20, 47.79) | 0.810 | -5.89 (-58.24, 46.46) | 0.825 |  |
| Family history of  chronic lung disease | No | -4.60 (-6.20, -3.00) | <0.001 | 0.0 (Ref.) | -18.26 (-33.15, -3.36) | 0.016 | -44.85 (-60.17, -29.53) | <0.001 | 0.017 |
|  | Yes | 0.06 (-3.43, 3.56) | 0.972 | 0.0 (Ref.) | -17.94 (-51.96, 16.08) | 0.301 | -6.25 (-39.92, 27.43) | 0.716 |  |
| NO_2_ | Low | -3.56 (-5.58, -1.53) | <0.001 | 0.0 (Ref.) | -16.98 (-35.65, 1.69) | 0.075 | -39.34 (-58.51, -20.17) | <0.001 | 0.453 |
|  | High | -4.21 (-6.34, -2.09) | <0.001 | 0.0 (Ref.) | -19.01 (-39.38, 1.35) | 0.067 | -38.88 (-59.57, -18.19) | <0.001 |  |
| NO_X_ | Low | -3.14 (-5.19, -1.08) | 0.003 | 0.0 (Ref.) | -19.54 (-38.39, -0.69) | 0.042 | -36.67 (-56.18, -17.15) | <0.001 | 0.185 |
|  | High | -4.56 (-6.65, -2.46) | <0.001 | 0.0 (Ref.) | -16.22 (-36.36, 3.93) | 0.115 | -41.31 (-61.60, -21.02) | <0.001 |  |
| PM_2.5_ | Low | -2.98 (-5.13, -0.83) | 0.007 | 0.0 (Ref.) | -20.30 (-40.03, -0.58) | 0.044 | -34.72 (-54.91, -14.53) | <0.001 | 0.265 |
|  | High | -4.48 (-6.59, -2.38) | <0.001 | 0.0 (Ref.) | -16.37 (-36.54, 3.79) | 0.112 | -39.94 (-60.52, -19.35) | <0.001 |  |
| PM_10_ | Low | -3.56 (-5.67, -1.44) | 0.001 | 0.0 (Ref.) | -20.61 (-40.22, -1.01) | 0.039 | -38.20 (-58.29, -18.11) | <0.001 | 0.533 |
|  | High | -3.96 (-6.10, -1.81) | <0.001 | 0.0 (Ref.) | -16.22 (-36.52, 4.08) | 0.117 | -35.78 (-56.48, -15.08) | <0.001 |  |
| PM_2.5-10_ | Low | -3.88 (-6.01, -1.75) | <0.001 | 0.0 (Ref.) | -24.86 (-44.62, -5.11) | 0.014 | -38.24 (-58.70, -17.78) | <0.001 | 0.712 |
|  | High | -3.63 (-5.76, -1.51) | <0.001 | 0.0 (Ref.) | -12.17 (-32.29, 7.95) | 0.236 | -35.50 (-55.81, -15.19) | <0.001 |  |

Abbreviations: NO_2_, nitrogen dioxide; NO_X_, nitrogen oxides; PM_2.5_, particulate matter with a diameter ≤2.5 μm; PM_10_, particulate matter with a diameter ≤10 μm

Model was adjusted age, sex, ethnicity (White, Mixed, South Asian, Black, Chinese, other races, or missing), educational attainment (no relevant qualifications, college or university degree, A levels/AS levels or equivalent, O levels/GCSEs or equivalent, CSEs or equivalent, others, or missing), socioeconomic deprivation index (quintiles, or missing), body mass index (<18.5, 18.5 to <25, 25 to <30, ≥30 kg/m^2,^ or missing), smoking status (never, previous, current, or missing), alcohol drinking (never, previous, current, or missing), healthy diet (yes, no, or missing), leisure time physical activity (<500, 500 to <1000, ≥1000 MET mins/week, or missing), family history of lung diseases (yes, no, or missing), diabetes (yes, no, or missing), air pollutants (NO_2_ [quintiles, or missing], NO_X_ [quintiles, or missing], PM_2.5_ [quintiles, or missing], PM_10_ [quintiles, or missing], PM_2.5-10_ [quintiles, or missing]).

**Supplementary Table S4.** Sensitivity analysis of the association between artificial sweetener intake and incident COPD risk, excluding participants with follow-up duration shorter than 3 or 5 years.

|  | Per 1-teaspoon increase in artificial sweeteners | | Non-consumers | Lower consumers | | Higher consumers |  |
| --- | --- | --- | --- | --- | --- | --- | --- |
|  | HR (95% CI) | P-value |  | HR (95% CI) | P-value | HR (95% CI) | P-value |
| Excluding follow-up duration<3 years |  |  |  |  |  |  |  |
| Events/total | 3041/161,815 |  | 2521/145,195 | 205/8524 |  | 315/8096 |  |
| Crude | 1.08 (1.07, 1.09) | <0.001 | 1.0 (Ref.) | 1.42 (1.25, 1.62) | <0.001 | 2.34 (2.10, 2.60) | <0.001 |
| Model 1 | 1.06 (1.05, 1.07) | <0.001 | 1.0 (Ref.) | 1.18 (1.03, 1.34) | 0.015 | 1.74 (1.56, 1.94) | <0.001 |
| Model 2 | 1.03 (1.02, 1.04) | <0.001 | 1.0 (Ref.) | 1.07 (0.94, 1.22) | 0.326 | 1.31 (1.17, 1.46) | <0.001 |
| Model 3 | 1.03 (1.02, 1.04) | <0.001 | 1.0 (Ref.) | 1.07 (0.94, 1.22) | 0.330 | 1.31 (1.18, 1.46) | <0.001 |
| Excluding follow-up duration<5 years |  |  |  |  |  |  |  |
| Events/total | 3599/163,540 |  | 2973/146,671 | 246/8640 |  | 380/8229 |  |
| Crude | 1.08 (1.07, 1.09) | <0.001 | 1.0 (Ref.) | 1.40 (1.21, 1.61) | <0.001 | 2.30 (2.04, 2.58) | <0.001 |
| Model 1 | 1.06 (1.05, 1.07) | <0.001 | 1.0 (Ref.) | 1.16 (1.01, 1.34) | 0.041 | 1.72 (1.53, 1.94) | <0.001 |
| Model 2 | 1.03 (1.01, 1.04) | <0.001 | 1.0 (Ref.) | 1.05 (0.91, 1.21) | 0.494 | 1.29 (1.14, 1.45) | <0.001 |
| Model 3 | 1.03 (1.01, 1.04) | <0.001 | 1.0 (Ref.) | 1.05 (0.91, 1.21) | 0.493 | 1.29 (1.15, 1.46) | <0.001 |

Model 1 was adjusted age, sex, ethnicity (White, Mixed, South Asian, Black, Chinese, other races, or missing), educational attainment (no relevant qualifications, college or university degree, A levels/AS levels or equivalent, O levels/GCSEs or equivalent, CSEs or equivalent, others, or missing), and socioeconomic deprivation index (quintiles, or missing); Model 2 was further adjusted for body mass index (<18.5, 18.5 to <25, 25 to <30, ≥30 kg/m^2,^ or missing), smoking status (never, previous, current, or missing), alcohol drinking (never, previous, current, or missing), healthy diet (yes, no, or missing), leisure time physical activity (<500, 500 to <1000, ≥1000 MET mins/week, or missing), family history of lung diseases (yes, no, or missing), diabetes (yes, no, or missing); Model 3 was further adjusted for air pollutants (NO_2_ [quintiles, or missing], NO_X_ [quintiles, or missing], PM_2.5_ [quintiles, or missing], PM_10_ [quintiles, or missing], PM_2.5-10_ [quintiles, or missing]).

**Supplementary Table S5.** Subgroup of the association between artificial sweetener intake and incident COPD risk, excluding participants with follow-up duration shorter than 3 years.

|  | Per 1-teaspoon increase in artificial sweeteners | | Non-consumers | Lower consumers | | Higher consumers |  |
| --- | --- | --- | --- | --- | --- | --- | --- |
|  | HR (95% CI) | P-value |  | HR (95% CI) | P-value | HR (95% CI) | P-value |
| Body mass index |  |  |  |  |  |  |  |
| <30 kg/m^2^ | 1.03 (1.02, 1.05) | <0.0001 | 1.0 (Ref.) | 1.03 (0.87, 1.21) | 0.7503 | 1.32 (1.15, 1.51) | <0.0001 |
| ≥30 kg/m^2^ | 1.02 (1.00, 1.04) | 0.0175 | 1.0 (Ref.) | 1.08 (0.86, 1.34) | 0.5070 | 1.28 (1.07, 1.54) | 0.0085 |

Model was adjusted age, sex, ethnicity (White, Mixed, South Asian, Black, Chinese, other races, or missing), educational attainment (no relevant qualifications, college or university degree, A levels/AS levels or equivalent, O levels/GCSEs or equivalent, CSEs or equivalent, others, or missing), socioeconomic deprivation index (quintiles, or missing), body mass index (<18.5, 18.5 to <25, 25 to <30, ≥30 kg/m^2,^ or missing), smoking status (never, previous, current, or missing), alcohol drinking (never, previous, current, or missing), healthy diet (yes, no, or missing), leisure time physical activity (<500, 500 to <1000, ≥1000 MET mins/week, or missing), family history of lung diseases (yes, no, or missing), diabetes (yes, no, or missing), air pollutants (NO_2_ [quintiles, or missing], NO_X_ [quintiles, or missing], PM_2.5_ [quintiles, or missing], PM_10_ [quintiles, or missing], PM_2.5-10_ [quintiles, or missing]).

**Supplementary Table S6.** The estimated mediation proportions for identified metabolites on the associations of artificial sweeteners with COPD.

|  | Average causal mediation effect | | Average direct effect | | Proportion mediated | |
| --- | --- | --- | --- | --- | --- | --- |
|  | OR (95% CI) | *P*_FDR_ | OR (95% CI) | *P*_FDR_ | % (95% CI) | *P*_FDR_ |
| Lipoprotein Particle Concentrations |  |  |  |  |  |  |
| Chylomicrons And Extremely Large VLDL Particles | -0.002 (-0.073, 0.067) | 0.985 | -16.177 (-26.726, -6.277) | <0.001 | 0.00% (-0.48%, 0.54%) | 0.985 |
| Very Large VLDL Particles | -0.009 (-0.118, 0.083) | 0.948 | -16.277 (-26.228, -7.055) | <0.001 | 0.02% (-0.63%, 0.90%) | 0.948 |
| Large VLDL Particles | -0.052 (-0.224, 0.078) | 0.684 | -16.168 (-25.702, -7.229) | <0.001 | 0.28% (-0.54%, 1.61%) | 0.684 |
| Medium VLDL Particles | -0.694 (-1.091, -0.332) | <0.001 | -15.173 (-24.717, -5.979) | 0.002 | 4.33% (1.95%, 10.92%) | <0.001 |
| Small VLDL Particles | -0.290 (-0.595, -0.085) | <0.001 | -15.648 (-26.272, -6.314) | <0.001 | 1.72% (0.53%, 5.14%) | <0.001 |
| Very Small VLDL Particles | -0.478 (-0.835, -0.175) | <0.001 | -15.716 (-25.388, -7.039) | <0.001 | 2.96% (1.05%, 7.11%) | <0.001 |
| IDL Particles | -1.042 (-1.627, -0.570) | <0.001 | -14.781 (-25.458, -6.360) | 0.002 | 6.52% (3.12%, 14.78%) | 0.006 |
| Large LDL Particles | -1.058 (-1.631, -0.617) | <0.001 | -14.661 (-24.136, -5.784) | 0.002 | 6.68% (3.42%, 16.56%) | 0.006 |
| Medium LDL Particles | -1.020 (-1.560, -0.610) | <0.001 | -14.948 (-25.005, -5.788) | 0.002 | 6.38% (3.31%, 15.21%) | 0.006 |
| Small LDL Particles | -0.926 (-1.388, -0.516) | <0.001 | -15.025 (-25.125, -5.700) | 0.006 | 5.72% (2.96%, 13.52%) | <0.001 |
| Very Large HDL Particles | -0.291 (-0.619, -0.046) | <0.001 | -15.875 (-25.108, -6.855) | <0.001 | 1.74% (0.29%, 5.40%) | <0.001 |
| Large HDL Particles | -0.088 (-0.375, 0.188) | 0.794 | -15.937 (-26.270, -6.464) | <0.001 | 0.54% (-1.27%, 3.02%) | 0.791 |
| Medium HDL Particles | -0.039 (-0.310, 0.243) | 0.917 | -16.039 (-26.443, -6.858) | <0.001 | 0.24% (-1.59%, 2.28%) | 0.915 |
| Small HDL Particles | -0.370 (-0.725, -0.091) | <0.001 | -15.457 (-25.402, -6.507) | 0.004 | 2.25% (0.52%, 6.68%) | 0.006 |
| Cholesterol Concentrations |  |  |  |  |  |  |
| Chylomicrons And Extremely Large VLDL | 0.014 (-0.052, 0.100) | 0.898 | -16.229 (-26.819, -6.917) | <0.001 | -0.04% (-0.77%, 0.34%) | 0.898 |
| Very Large VLDL Particles | -0.119 (-0.310, 0.022) | 0.227 | -15.977 (-25.436, -6.803) | <0.001 | 0.70% (-0.17%, 2.46%) | 0.227 |
| Large VLDL Particles | -0.136 (-0.335, 0.021) | 0.200 | -15.802 (-26.129, -6.912) | <0.001 | 0.83% (-0.12%, 2.70%) | 0.200 |
| Medium VLDL Particles | -1.048 (-1.578, -0.622) | <0.001 | -14.392 (-24.105, -5.778) | <0.001 | 6.77% (3.39%, 16.24%) | <0.001 |
| Small VLDL Particles | -0.647 (-1.071, -0.318) | <0.001 | -15.154 (-24.732, -5.679) | <0.001 | 4.05% (1.81%, 10.99%) | <0.001 |
| Very Small VLDL Particles | -0.733 (-1.189, -0.360) | <0.001 | -15.227 (-25.332, -5.754) | <0.001 | 4.54% (2.20%, 11.20%) | <0.001 |
| IDL Particles | -1.374 (-1.996, -0.858) | <0.001 | -14.270 (-24.539, -5.152) | 0.002 | 8.64% (4.64%, 21.90%) | 0.006 |
| Large LDL Particles | -0.686 (-0.981, -0.436) | <0.001 | -15.137 (-25.171, -6.068) | <0.001 | 4.34% (2.34%, 10.56%) | <0.001 |
| Medium LDL Particles | -0.995 (-1.536, -0.600) | <0.001 | -14.838 (-24.217, -5.382) | 0.002 | 6.22% (3.35%, 14.90%) | 0.006 |
| Small LDL Particles | -0.950 (-1.392, -0.580) | <0.001 | -14.647 (-24.633, -4.860) | 0.002 | 6.08% (3.29%, 14.33%) | 0.006 |
| Very Large HDL Particles | -0.222 (-0.502, -0.001) | 0.100 | -15.755 (-26.457, -6.108) | <0.001 | 1.35% (0.01%, 4.41%) | 0.100 |
| Large HDL Particles | -0.044 (-0.312, 0.195) | 0.907 | -15.934 (-26.071, -6.618) | <0.001 | 0.27% (-1.52%, 2.30%) | 0.905 |
| Medium HDL Particles | -0.025 (-0.317, 0.251) | 0.932 | -16.160 (-26.911, -7.344) | <0.001 | 0.14% (-1.74%, 2.39%) | 0.932 |
| Small HDL Particles | -0.351 (-0.703, -0.066) | <0.001 | -15.656 (-24.794, -7.371) | 0.002 | 2.13% (0.38%, 6.00%) | <0.001 |
| Free Cholesterol Concentrations |  |  |  |  |  |  |
| Chylomicrons And Extremely Large VLDL Particles | 0.010 (-0.066, 0.117) | 0.917 | -16.310 (-26.692, -6.782) | <0.001 | -0.02% (-0.80%, 0.41%) | 0.917 |
| Very Large VLDL Particles | -0.044 (-0.196, 0.083) | 0.704 | -16.259 (-27.072, -6.463) | <0.001 | 0.24% (-0.60%, 1.44%) | 0.704 |
| Large VLDL Particles | -0.073 (-0.258, 0.062) | 0.481 | -15.839 (-25.844, -6.274) | <0.001 | 0.40% (-0.41%, 2.07%) | 0.481 |
| Medium VLDL Particles | -0.839 (-1.323, -0.475) | <0.001 | -14.637 (-24.218, -5.785) | 0.002 | 5.46% (2.75%, 13.14%) | <0.001 |
| Small VLDL Particles | -0.870 (-1.386, -0.462) | <0.001 | -14.854 (-24.327, -5.958) | 0.002 | 5.53% (2.77%, 12.87%) | <0.001 |
| Very Small VLDL Particles | -0.480 (-0.846, -0.189) | <0.001 | -15.240 (-24.630, -6.085) | 0.006 | 3.05% (1.11%, 7.82%) | 0.011 |
| IDL Particles | -1.236 (-1.799, -0.732) | <0.001 | -14.259 (-24.062, -5.422) | 0.004 | 8.06% (4.18%, 19.32%) | 0.006 |
| Large LDL Particles | -0.833 (-1.203, -0.538) | <0.001 | -15.088 (-25.666, -5.989) | 0.002 | 5.20% (2.92%, 12.86%) | <0.001 |
| Medium LDL Particles | -0.836 (-1.211, -0.515) | <0.001 | -14.803 (-24.586, -5.572) | 0.002 | 5.29% (2.85%, 12.80%) | <0.001 |
| Small LDL Particles | -0.704 (-1.059, -0.431) | <0.001 | -14.689 (-24.360, -5.483) | 0.002 | 4.49% (2.48%, 11.74%) | 0.006 |
| Very Large HDL Particles | -0.151 (-0.352, 0.001) | 0.115 | -16.223 (-26.698, -7.340) | <0.001 | 0.90% (-0.01%, 2.83%) | 0.115 |
| Large HDL Particles | 0.009 (-0.217, 0.244) | 0.953 | -16.155 (-26.084, -6.836) | <0.001 | -0.07% (-1.98%, 1.47%) | 0.955 |
| Medium HDL Particles | -0.120 (-0.453, 0.177) | 0.704 | -16.011 (-26.119, -6.528) | <0.001 | 0.73% (-1.30%, 3.42%) | 0.704 |
| Small HDL Particles | -0.397 (-0.822, -0.058) | <0.001 | -15.692 (-26.248, -6.769) | 0.002 | 2.48% (0.37%, 7.18%) | 0.006 |
| Esterified Cholesterol Concentrations |  |  |  |  |  |  |
| Chylomicrons And Extremely Large VLDL Particles | 0.014 (-0.061, 0.114) | 0.907 | -16.062 (-26.793, -6.635) | 0.002 | -0.04% (-0.87%, 0.42%) | 0.905 |
| Very Large VLDL Particles | -0.200 (-0.452, -0.033) | <0.001 | -15.792 (-25.715, -6.672) | 0.004 | 1.22% (0.19%, 3.64%) | <0.001 |
| Large VLDL Particles | -0.230 (-0.490, -0.041) | <0.001 | -15.867 (-25.276, -6.588) | <0.001 | 1.37% (0.24%, 4.13%) | <0.001 |
| Medium VLDL Particles | -0.743 (-1.148, -0.374) | <0.001 | -14.698 (-24.333, -5.717) | 0.002 | 4.78% (2.23%, 12.42%) | 0.006 |
| Small VLDL Particles | -0.511 (-0.928, -0.229) | <0.001 | -15.500 (-25.180, -6.237) | 0.002 | 3.15% (1.39%, 8.04%) | 0.006 |
| Very Small VLDL Particles | -0.846 (-1.339, -0.456) | <0.001 | -14.927 (-24.526, -6.597) | <0.001 | 5.42% (2.36%, 11.93%) | <0.001 |
| IDL Particles | -1.369 (-2.042, -0.836) | <0.001 | -14.300 (-24.003, -5.469) | <0.001 | 8.68% (4.78%, 20.58%) | <0.001 |
| Large LDL Particles | -0.670 (-0.981, -0.411) | 0.006 | -15.496 (-26.640, -6.265) | 0.002 | 4.20% (2.37%, 9.59%) | 0.011 |
| Medium LDL Particles | -0.918 (-1.407, -0.540) | <0.001 | -14.753 (-25.094, -5.531) | <0.001 | 5.86% (2.99%, 14.44%) | <0.001 |
| Small LDL Particles | -0.906 (-1.354, -0.541) | <0.001 | -14.540 (-24.765, -5.706) | 0.002 | 5.80% (3.08%, 14.59%) | 0.006 |
| Very Large HDL Particles | -0.084 (-0.273, 0.083) | 0.565 | -15.967 (-26.197, -6.739) | <0.001 | 0.48% (-0.58%, 2.32%) | 0.565 |
| Large HDL Particles | -0.055 (-0.303, 0.185) | 0.896 | -16.147 (-26.954, -6.215) | <0.001 | 0.32% (-1.41%, 2.53%) | 0.896 |
| Medium HDL Particles | -0.021 (-0.300, 0.240) | 0.948 | -16.000 (-26.276, -6.209) | <0.001 | 0.14% (-1.67%, 2.36%) | 0.948 |
| Small HDL Particles | -0.304 (-0.636, -0.040) | 0.054 | -15.578 (-25.441, -6.765) | <0.001 | 1.88% (0.28%, 5.18%) | 0.054 |
| Triglyceride Concentrations |  |  |  |  |  |  |
| Chylomicrons And Extremely Large VLDL Particles | -0.011 (-0.107, 0.054) | 0.914 | -16.051 (-26.465, -6.952) | <0.001 | 0.03% (-0.36%, 0.74%) | 0.914 |
| Very Large VLDL Particles | 0.010 (-0.065, 0.107) | 0.930 | -16.054 (-26.076, -6.618) | <0.001 | -0.03% (-0.74%, 0.48%) | 0.930 |
| Large VLDL Particles | -0.028 (-0.184, 0.110) | 0.900 | -16.037 (-25.832, -6.719) | 0.002 | 0.16% (-0.82%, 1.29%) | 0.903 |
| Medium VLDL Particles | -0.112 (-0.313, 0.036) | 0.305 | -16.046 (-25.522, -7.298) | <0.001 | 0.65% (-0.25%, 2.30%) | 0.305 |
| Small VLDL Particles | -0.023 (-0.130, 0.042) | 0.794 | -16.214 (-26.375, -6.868) | 0.002 | 0.09% (-0.32%, 0.92%) | 0.791 |
| Very Small VLDL Particles | 0.013 (-0.056, 0.103) | 0.907 | -15.855 (-26.074, -6.566) | 0.004 | -0.05% (-0.82%, 0.37%) | 0.905 |
| IDL Particles | 0.027 (-0.088, 0.169) | 0.898 | -16.201 (-26.654, -6.526) | <0.001 | -0.14% (-1.32%, 0.64%) | 0.898 |
| Large LDL Particles | 0.014 (-0.143, 0.173) | 0.932 | -15.955 (-25.116, -6.988) | <0.001 | -0.08% (-1.19%, 1.00%) | 0.932 |
| Medium LDL Particles | -0.032 (-0.177, 0.099) | 0.835 | -16.018 (-26.321, -6.743) | 0.002 | 0.18% (-0.68%, 1.25%) | 0.832 |
| Small LDL Particles | -0.037 (-0.155, 0.047) | 0.619 | -15.878 (-25.829, -7.057) | <0.001 | 0.18% (-0.32%, 1.17%) | 0.619 |
| Very Large HDL Particles | -0.133 (-0.368, 0.035) | 0.205 | -16.207 (-26.007, -6.949) | 0.002 | 0.78% (-0.21%, 2.79%) | 0.209 |
| Large HDL Particles | -0.014 (-0.195, 0.173) | 0.948 | -16.076 (-27.407, -7.285) | <0.001 | 0.07% (-1.28%, 1.36%) | 0.948 |
| Medium HDL Particles | 0.025 (-0.041, 0.139) | 0.794 | -16.252 (-26.091, -7.284) | <0.001 | -0.10% (-0.94%, 0.28%) | 0.791 |
| Small HDL Particles | -0.005 (-0.083, 0.055) | 0.948 | -16.231 (-26.788, -7.072) | <0.001 | 0.01% (-0.39%, 0.52%) | 0.948 |
| Phospholipids Concentrations |  |  |  |  |  |  |
| Chylomicrons And Extremely Large VLDL Particles | 0.011 (-0.065, 0.107) | 0.917 | -16.029 (-27.251, -6.443) | <0.001 | -0.03% (-0.74%, 0.46%) | 0.915 |
| Very Large VLDL Particles | -0.032 (-0.180, 0.084) | 0.838 | -15.999 (-26.362, -7.238) | <0.001 | 0.17% (-0.54%, 1.44%) | 0.838 |
| Large VLDL Particles | -0.031 (-0.160, 0.051) | 0.712 | -16.166 (-27.559, -6.351) | <0.001 | 0.13% (-0.37%, 1.03%) | 0.712 |
| Medium VLDL Particles | -0.565 (-0.919, -0.296) | <0.001 | -15.343 (-24.902, -5.943) | 0.002 | 3.51% (1.67%, 8.78%) | <0.001 |
| Small VLDL Particles | -0.633 (-1.077, -0.304) | <0.001 | -15.105 (-24.942, -5.535) | 0.002 | 4.02% (1.83%, 10.38%) | <0.001 |
| Very Small VLDL Particles | -0.263 (-0.541, -0.041) | <0.001 | -15.605 (-25.442, -5.447) | 0.002 | 1.58% (0.26%, 5.61%) | 0.006 |
| IDL Particles | -1.122 (-1.691, -0.620) | <0.001 | -14.443 (-24.739, -5.008) | <0.001 | 7.16% (3.72%, 17.50%) | <0.001 |
| Large LDL Particles | -1.328 (-2.011, -0.809) | <0.001 | -14.105 (-23.667, -5.652) | 0.004 | 8.72% (4.53%, 19.63%) | <0.001 |
| Medium LDL Particles | -1.070 (-1.634, -0.605) | <0.001 | -14.418 (-24.137, -4.752) | <0.001 | 6.82% (3.62%, 18.72%) | <0.001 |
| Small LDL Particles | -0.935 (-1.441, -0.562) | <0.001 | -14.619 (-24.204, -5.513) | <0.001 | 5.97% (3.04%, 14.15%) | <0.001 |
| Very Large HDL Particles | -0.025 (-0.129, 0.057) | 0.794 | -16.206 (-26.151, -7.206) | <0.001 | 0.12% (-0.35%, 0.99%) | 0.791 |
| Large HDL Particles | 0.038 (-0.218, 0.307) | 0.907 | -16.041 (-27.280, -6.045) | 0.002 | -0.22% (-2.55%, 1.41%) | 0.905 |
| Medium HDL Particles | 0.095 (-0.122, 0.365) | 0.653 | -16.215 (-26.331, -6.448) | <0.001 | -0.58% (-2.70%, 0.87%) | 0.653 |
| Small HDL Particles | -0.029 (-0.254, 0.186) | 0.917 | -16.055 (-26.323, -6.830) | 0.004 | 0.15% (-1.20%, 1.76%) | 0.915 |
| Total Lipid Concentrations |  |  |  |  |  |  |
| Chylomicrons And Extremely Large VLDL Particles | 0.013 (-0.056, 0.105) | 0.907 | -16.239 (-26.485, -7.391) | <0.001 | -0.04% (-0.70%, 0.40%) | 0.905 |
| Very Large VLDL Particles | -0.012 (-0.125, 0.087) | 0.923 | -16.102 (-26.167, -7.133) | <0.001 | 0.05% (-0.61%, 0.85%) | 0.923 |
| Large VLDL Particles | -0.063 (-0.233, 0.085) | 0.619 | -16.003 (-26.355, -7.327) | <0.001 | 0.34% (-0.51%, 1.80%) | 0.619 |
| Medium VLDL Particles | -0.536 (-0.902, -0.233) | <0.001 | -15.075 (-25.012, -6.030) | <0.001 | 3.43% (1.42%, 8.34%) | <0.001 |
| Small VLDL Particles | -0.340 (-0.623, -0.116) | <0.001 | -15.416 (-24.920, -6.641) | 0.002 | 2.10% (0.72%, 5.82%) | 0.006 |
| Very Small VLDL Particles | -0.407 (-0.753, -0.115) | <0.001 | -15.568 (-25.000, -6.418) | 0.002 | 2.50% (0.66%, 6.59%) | 0.006 |
| IDL Particles | -1.264 (-1.850, -0.736) | 0.011 | -14.528 (-25.494, -5.563) | <0.001 | 7.94% (4.17%, 18.34%) | 0.011 |
| Large LDL Particles | -1.350 (-1.973, -0.842) | <0.001 | -14.377 (-24.523, -5.425) | 0.002 | 8.58% (4.60%, 20.21%) | 0.006 |
| Medium LDL Particles | -1.066 (-1.593, -0.639) | 0.006 | -14.668 (-25.280, -6.165) | <0.001 | 6.86% (3.55%, 15.63%) | 0.006 |
| Small LDL Particles | -1.038 (-1.563, -0.592) | <0.001 | -14.351 (-24.604, -5.298) | <0.001 | 6.81% (3.47%, 16.51%) | <0.001 |
| Very Large HDL Particles | -0.121 (-0.355, 0.076) | 0.397 | -16.032 (-25.529, -6.986) | <0.001 | 0.71% (-0.43%, 2.75%) | 0.397 |
| Large HDL Particles | -0.034 (-0.294, 0.226) | 0.917 | -16.171 (-26.689, -6.363) | 0.002 | 0.20% (-1.55%, 2.18%) | 0.915 |
| Medium HDL Particles | 0.048 (-0.214, 0.324) | 0.907 | -16.056 (-26.064, -6.229) | <0.001 | -0.30% (-2.25%, 1.45%) | 0.905 |
| Small HDL Particles | -0.127 (-0.383, 0.091) | 0.463 | -15.792 (-25.529, -7.015) | <0.001 | 0.77% (-0.58%, 2.95%) | 0.463 |
| Lipoprotein Particle Sizes |  |  |  |  |  |  |
| Average Diameter For VLDL Particles | 0.072 (-0.019, 0.229) | 0.294 | -15.982 (-26.358, -6.689) | <0.001 | -0.40% (-1.74%, 0.14%) | 0.294 |
| Average Diameter For LDL Particles | -0.081 (-0.235, 0.014) | 0.228 | -16.068 (-26.569, -6.601) | 0.002 | 0.45% (-0.11%, 1.93%) | 0.232 |
| Average Diameter For HDL Particles | 0.027 (-0.167, 0.234) | 0.917 | -16.329 (-26.382, -6.906) | <0.001 | -0.14% (-1.61%, 1.23%) | 0.915 |
| Apolipoprotein A1 | -0.127 (-0.458, 0.189) | 0.704 | -15.961 (-26.686, -6.556) | 0.002 | 0.76% (-1.35%, 3.76%) | 0.704 |
| Apolipoprotein B | -1.057 (-1.655, -0.641) | 0.011 | -14.478 (-24.716, -5.481) | 0.004 | 6.70% (3.67%, 16.35%) | 0.021 |
| Apolipoprotein B To Apolipoprotein A1 Ratio | -0.306 (-0.597, -0.086) | 0.050 | -15.546 (-26.385, -6.702) | <0.001 | 1.87% (0.53%, 4.99%) | 0.050 |
| Fatty Acids |  |  |  |  |  |  |
| Polyunsaturated | -1.032 (-1.602, -0.583) | 0.011 | -14.510 (-24.514, -5.325) | <0.001 | 6.55% (3.35%, 18.14%) | 0.011 |
| Monounsaturated | 0.001 (-0.125, 0.130) | 0.992 | -16.199 (-25.818, -7.302) | <0.001 | 0.00% (-0.96%, 0.85%) | 0.992 |
| Saturated | -0.129 (-0.356, 0.068) | 0.367 | -15.842 (-25.920, -6.763) | <0.001 | 0.78% (-0.50%, 2.94%) | 0.367 |
| Docosahexaenoic Acid | -0.579 (-0.984, -0.269) | 0.043 | -15.034 (-24.977, -6.207) | <0.001 | 3.70% (1.58%, 9.50%) | 0.043 |
| Linoleic Acid | -1.149 (-1.705, -0.690) | 0.006 | -14.710 (-24.185, -5.869) | <0.001 | 7.16% (3.92%, 16.47%) | 0.006 |
| Omega-3 | -0.319 (-0.644, -0.046) | 0.043 | -15.539 (-24.768, -6.494) | <0.001 | 1.99% (0.32%, 5.56%) | 0.043 |
| Omega-6 | -0.931 (-1.420, -0.530) | 0.047 | -14.839 (-25.270, -5.893) | <0.001 | 5.91% (2.94%, 15.28%) | 0.047 |
| Total | -0.302 (-0.606, -0.069) | 0.026 | -15.294 (-25.297, -5.950) | 0.002 | 1.92% (0.36%, 5.35%) | 0.031 |
| Polyunsaturated To Total Fatty Acids Ratio | -0.198 (-0.457, 0.008) | 0.141 | -15.981 (-25.800, -7.110) | <0.001 | 1.19% (-0.05%, 3.64%) | 0.141 |
| MonounsaturatedTo Total Fatty Acids Ratio | -0.331 (-0.669, -0.061) | <0.001 | -15.927 (-26.155, -6.609) | 0.002 | 2.03% (0.35%, 5.21%) | 0.006 |
| SaturatedTo Total Fatty Acids Ratio | 0.041 (-0.176, 0.290) | 0.912 | -16.073 (-26.589, -7.063) | <0.001 | -0.21% (-2.15%, 1.25%) | 0.912 |
| Docosahexaenoic Acid To Total Fatty Acids Ratio | -0.137 (-0.408, 0.078) | 0.373 | -15.840 (-25.851, -7.386) | <0.001 | 0.83% (-0.55%, 3.08%) | 0.373 |
| Linoleic Acid To Total Fatty Acids Ratio | -0.789 (-1.223, -0.408) | <0.001 | -15.003 (-23.954, -6.356) | 0.004 | 4.96% (2.42%, 11.39%) | 0.006 |
| Omega-3 To Total Fatty Acids Ratio | 0.022 (-0.243, 0.292) | 0.948 | -15.999 (-26.250, -7.423) | <0.001 | -0.10% (-2.42%, 1.53%) | 0.948 |
| Omega-6 To Total Fatty Acids Ratio | -0.118 (-0.316, 0.021) | 0.211 | -16.095 (-26.415, -7.166) | <0.001 | 0.68% (-0.15%, 2.37%) | 0.211 |
| Other lipids |  |  |  |  |  |  |
| Total Cholines | -0.716 (-1.258, -0.306) | 0.050 | -15.267 (-25.437, -6.530) | 0.002 | 4.42% (1.88%, 11.16%) | 0.050 |
| Phosphatidylcholines | -0.686 (-1.138, -0.269) | <0.001 | -15.629 (-25.375, -6.773) | <0.001 | 4.17% (1.60%, 10.45%) | <0.001 |
| Sphingomyelins | -0.743 (-1.254, -0.291) | 0.039 | -14.976 (-25.475, -5.520) | <0.001 | 4.77% (1.77%, 14.22%) | 0.039 |
| Phosphoglycerides | -0.548 (-1.043, -0.185) | 0.031 | -15.378 (-25.516, -5.910) | <0.001 | 3.42% (1.05%, 10.22%) | 0.031 |
| Glycolysis-related Metabolities | 0.000 (0.000, 0.000) |  | 0.000 (0.000, 0.000) |  | 0.00% (0.00%, 0.00%) |  |
| Lactate | 0.000 (-0.158, 0.144) | 0.985 | -16.420 (-26.416, -6.900) | <0.001 | -0.01% (-1.09%, 1.18%) | 0.985 |
| Citrate | -0.309 (-0.594, -0.086) | 0.039 | -15.839 (-26.712, -6.707) | <0.001 | 1.88% (0.50%, 5.18%) | 0.039 |
| Glucose | -0.017 (-0.147, 0.084) | 0.907 | -16.379 (-26.457, -7.010) | <0.001 | 0.07% (-0.62%, 1.09%) | 0.905 |
| Amino Acids |  |  |  |  |  |  |
| Alanine | 0.028 (-0.164, 0.229) | 0.917 | -16.134 (-26.091, -7.045) | <0.001 | -0.15% (-2.02%, 1.10%) | 0.915 |
| Glutamine | -0.023 (-0.220, 0.172) | 0.917 | -16.384 (-26.640, -7.109) | <0.001 | 0.12% (-1.15%, 1.52%) | 0.915 |
| Histidine | 0.006 (-0.197, 0.209) | 0.962 | -16.454 (-26.615, -7.453) | <0.001 | -0.05% (-1.44%, 1.47%) | 0.962 |
| Glycine | 0.070 (-0.011, 0.211) | 0.263 | -16.384 (-27.048, -7.096) | <0.001 | -0.38% (-1.65%, 0.07%) | 0.263 |
| Isoleucine | -0.013 (-0.109, 0.053) | 0.907 | -15.681 (-25.393, -6.520) | <0.001 | 0.04% (-0.39%, 0.80%) | 0.905 |
| Leucine | 0.011 (-0.130, 0.148) | 0.932 | -15.750 (-25.532, -6.406) | <0.001 | -0.05% (-1.31%, 0.94%) | 0.932 |
| Valine | -0.067 (-0.250, 0.092) | 0.638 | -15.212 (-25.543, -6.160) | <0.001 | 0.42% (-0.62%, 2.04%) | 0.638 |
| Phenylalanine | -0.003 (-0.077, 0.069) | 0.953 | -16.154 (-26.273, -7.409) | <0.001 | 0.01% (-0.47%, 0.54%) | 0.954 |
| Tyrosine | 0.044 (-0.044, 0.178) | 0.558 | -16.244 (-25.776, -7.177) | <0.001 | -0.24% (-1.33%, 0.24%) | 0.558 |
| Ketone bodies |  |  |  |  |  |  |
| Acetate | -0.040 (-0.190, 0.069) | 0.696 | -16.254 (-26.305, -7.066) | <0.001 | 0.20% (-0.45%, 1.42%) | 0.696 |
| Acetoacetate | -0.007 (-0.113, 0.096) | 0.953 | -16.286 (-26.139, -6.574) | 0.002 | 0.01% (-0.66%, 0.77%) | 0.955 |
| 3 Hydroxybutyrate | 0.033 (-0.061, 0.182) | 0.806 | -16.458 (-26.630, -7.572) | <0.001 | -0.14% (-1.29%, 0.48%) | 0.806 |
| Acetone | 0.021 (-0.037, 0.118) | 0.838 | -16.242 (-26.251, -7.196) | <0.001 | -0.08% (-0.88%, 0.28%) | 0.838 |
| Pyruvate | 0.018 (-0.053, 0.115) | 0.872 | -16.376 (-25.982, -7.405) | 0.002 | -0.06% (-0.86%, 0.37%) | 0.875 |
| Fluid balance |  |  |  |  |  |  |
| Albumin | -0.380 (-0.767, -0.024) | 0.070 | -15.829 (-26.480, -6.456) | <0.001 | 2.34% (0.13%, 6.38%) | 0.070 |
| Creatinine | -0.019 (-0.116, 0.047) | 0.869 | -16.330 (-25.695, -7.097) | <0.001 | 0.07% (-0.31%, 0.82%) | 0.869 |
| Inflammation |  |  |  |  |  |  |
| Glycoprotein Acetyls | -0.300 (-0.671, 0.026) | 0.139 | -16.002 (-26.218, -6.444) | 0.002 | 1.74% (-0.22%, 5.72%) | 0.141 |

Mediation analyses were adjusted for age, sex, ethnicity (White, Mixed, South Asian, Black, Chinese, other races, or missing), educational attainment (no relevant qualifications, college or university degree, A levels/AS levels or equivalent, O levels/GCSEs or equivalent, CSEs or equivalent, others, or missing), socioeconomic deprivation index (quintiles, or missing), body mass index (<18.5, 18.5 to <25, 25 to <30, ≥30 kg/m^2,^ or missing), smoking status (never, previous, current, or missing), alcohol drinking (never, previous, current, or missing), healthy diet (yes, no, or missing), leisure time physical activity (<500, 500 to <1000, ≥1000 MET mins/week, or missing), family history of lung diseases (yes, no, or missing), diabetes (yes, no, or missing), air pollutants (NO_2_ [quintiles, or missing], NO_X_ [quintiles, or missing], PM_2.5_ [quintiles, or missing], PM_10_ [quintiles, or missing], PM_2.5-10_ [quintiles, or missing]). *P* values were based on two-sided tests. OR, odds ratio; CI, confidence interval;
